# Parent‐offspring inference in inbred populations

**DOI:** 10.1111/1755-0998.13680

**Published:** 2022-07-22

**Authors:** Jan‐Niklas Runge, Barbara König, Anna K. Lindholm, Andres Bendesky

**Affiliations:** ^1^ Department of Ecology, Evolution and Environmental Biology, Zuckerman Mind Brain Behavior Institute Columbia University New York NY USA; ^2^ Department of Evolutionary Biology and Environmental Studies University of Zurich Zürich Switzerland

**Keywords:** genotyping errors, homozygosity, identity by descent, inbreeding, pedigree reconstruction, relatedness

## Abstract

Genealogical relationships are fundamental components of genetic studies. However, it is often challenging to infer correct and complete pedigrees even when genome‐wide information is available. For example, inbreeding can obscure genetic differences between individuals, making it difficult to even distinguish first‐degree relatives such as parent‐offspring from full siblings. Similarly, genotyping errors can interfere with the detection of genetic similarity between parents and their offspring. Inbreeding is common in natural, domesticated, and experimental populations and genotyping of these populations often has more errors than in human data sets, so efficient methods for building pedigrees under these conditions are necessary. Here, we present a new method for parent‐offspring inference in inbred pedigrees called specific parent‐offspring relationship estimation (spore). spore is vastly superior to existing pedigree‐inference methods at detecting parent‐offspring relationships, in particular when inbreeding is high or in the presence of genotyping errors, or both. spore therefore fills an important void in the arsenal of pedigree inference tools.

## 1 INTRODUCTION

Genealogical relationships among individuals are critical for many genetic analyses. For example, in trait‐mapping studies based on linkage (Ott et al., 2015) and transmission disequilibrium analyses (e.g., Spielman & Ewens, 1998) as well as in sibling‐based genome‐wide association studies (Howe et al., 2021), the transmission of traits and alleles is analysed within pedigrees to control for environmental similarities and to avoid confounders from population structure. Pedigrees can also be fit into statistical models to estimate the heritabilities of traits (Kaplanis et al., 2018; Wilson et al., 2010). Removing close relatives from analyses can also be important to fulfil analytical assumptions in population genetics (Wang, 2004). Pedigrees are also useful to study the rate of de novo mutations and their impact on traits (Koch et al., 2019). Furthermore, pedigrees themselves can be of considerable interest, for example when analysing differences in lifetime reproductive success (Farquharson et al., 2017) or assortative mating (Grant & Grant, 2008). Thus, accurate and complete pedigree relationships are important to maximize power and accuracy of genetic analyses and for studies of population biology.

Deriving complete and correct pedigrees can be challenging. In monitored wild populations, researchers may use a combination of observed data, such as dates of birth and death, coupled with sparse genotypes produced by technologies such as microsatellites, and employ software tools such as colony (Wang, 2004) to build pedigrees. This approach is usually accurate, but has two major drawbacks. First, documenting dates of birth and death (to exclude impossible parent‐offspring matches) can be difficult in some species and contexts. Second, it is impractical to genotype large numbers of microsatellites, decreasing the power to infer genealogical relationships in populations with low genetic diversity.

**FIGURE 1:**
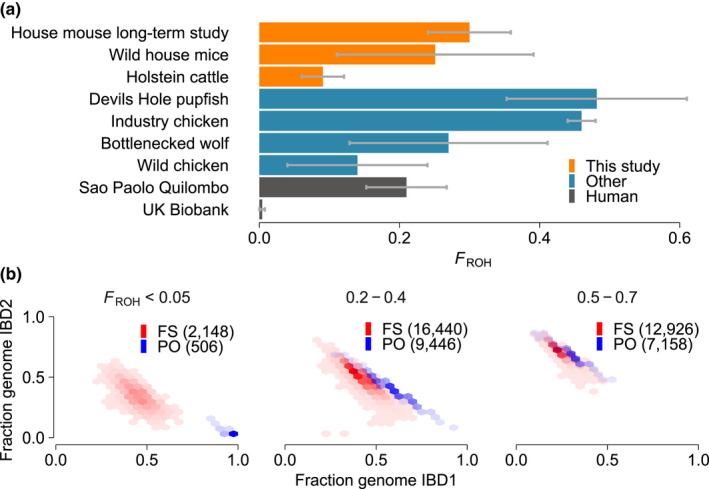
Extent of inbreeding in multiple populations and its influence on metrics used to infer relationships. (a) Overview of levels of inbreeding, measured as $F_{\mathrm{ROH}}$ (see Methods S1), in the populations studied here (orange) and in other nonhuman (blue) and human (grey) populations (Johnson et al., 2018; Kardos et al., 2018; Lemes et al., 2018; Talebi et al., 2020; Tian et al., 2021). Bars indicate average, error bars indicate the standard deviation, based on one population of the given group, with the exception of industry chicken (four groups). (b) Heatmaps of the fractions of genomes that are IBD1 and IBD2 in parent‐offspring (PO, blue) and full sibling (FS, red) relationships. Each plot corresponds to different $F_{\mathrm{ROH}}$ levels. Data is based on the simulated pedigrees with genotyping errors (see Methods S1), with 17,110 PO and 31,514 FS relationships.

Current genome‐wide methods for pedigree reconstruction are often developed and optimized for outbred human populations and thus compatibility with considerable levels of inbreeding is not the main concern (e.g., Huff et al., 2011). However, some human populations and individuals have high levels of inbreeding, with up to 20% of their genome empirically determined to be in a homozygous state (Ceballos et al., 2018; Keller et al., 2011; Yengo et al., 2019; Figure 1a). Considerable inbreeding is also present in populations of other species, including livestock (Alemu et al., 2021; Talebi et al., 2020; and Methods), small populations of wild animals such as wolves (Kardos et al., 2018) and Devils Hole pupfish (Tian et al., 2021), and even in populations with large census sizes such as house mice (Figure 1a). Inbreeding violates the assumptions of many genealogical‐relationship inferring software, yet the performance of these tools in the presence of inbreeding has not been thoroughly evaluated. Inbreeding homogenizes genomes within populations, making it difficult to leverage patterns of genetic variant sharing to distinguish different types of relatives. For example, the fractions of their genomes two individuals share identical‐by‐descent (“IBD,” inherited through a recent common ancestor), in only one of the two homologous chromosomes (“IBD1”) or in both homologous chromosomes (“IBD2”), differ between parent‐offspring and full‐sibling relationships. Hence, these metrics are often used to distinguish such relationships (Li et al., 2014; Staples et al., 2014; Stevens et al., 2011). However, when parents are genetically related, the fraction of the genome that is IBD2 with their offspring is higher than in outbred populations and is thus more similar to full‐sibling relationships (Figure 1b). Thus, inbreeding could hinder the accuracy of existing pedigree‐reconstruction tools.

Typical genotyping error levels in human studies can also have a large impact on pedigree inference (Smith et al., 2021; Wang, 2010) and these errors might be more prevalent in less well‐studied species and populations (Bresadola et al., 2020; Browning & Browning, 2013; Money et al., 2015). Genotyping errors can result in parent‐offspring relationships appearing as less related than expected, obscuring the distinction between parent‐offspring and other types of relationships. Furthermore, these errors can interrupt inferred IBD segments, limiting the utility of segment number and length for genealogical inference (Smith et al., 2021).

Here, we present a new method, which we call specific parent‐offspring relationship estimation (spore), that is robust to high levels of inbreeding and errors in genotyping. It is designed to use genome‐wide genotypes from a variety of sources, such as those derived from high‐coverage sequencing, imputation, or genotyping arrays. Instead of the commonly used IBD1 and IBD2, our method relies on related allele‐sharing metrics that have large expected differences between parent‐offspring and other types of relationships, even in highly inbred individuals. We show that spore is more sensitive and accurate than other commonly used methods that are not designed for inbred populations and that spore is more tolerant to genotyping errors, making it a robust tool for detecting relatives and building pedigrees using genetic data.

## 2 MATERIALS AND METHODS

We developed spore as a robust method for parent‐offspring inference and pedigree building in inbred populations and to be tolerant to genotyping errors. The main differences with existing methods are (1) the focus on variables that are expected to be zero in parent‐offspring relationships but higher in other relationships (with the exception of monozygotic twins) and (2) the automatic adjustment of thresholds below which a relationship is classified as parent‐offspring. This contrasts with other methods' use of fixed thresholds derived from theory assuming outbred populations (e.g., Manichaikul et al., 2010; Qiao et al., 2021), which can be violated by inbreeding and genotyping errors. Our approach also contrasts with coancestry, which simulates parent‐offspring relationships using the genetic information of the individuals in the data set, an approach that does not model genotyping errors nor resemble the distribution of inbreeding levels of the sample (Wang, 2011).

The essential elements of spore are described here, beginning with the variables that we use for finding parent‐offspring relationships, followed by a description of the algorithm. Additional details are described in the Supporting Information.

spore takes a VCF file as input and its primary output is a pedigree file with the first columns in PED‐compatible format. The IBD software we employ (truffle, Dimitromanolakis et al., 2019) requires ≥ 10,000 variants mapped to a genome reference. However, spore is modular and users can provide it with IBD values estimated through alternative methods.

### 2.1 Variables used to infer parent‐offspring relationships

**FIGURE 2:**
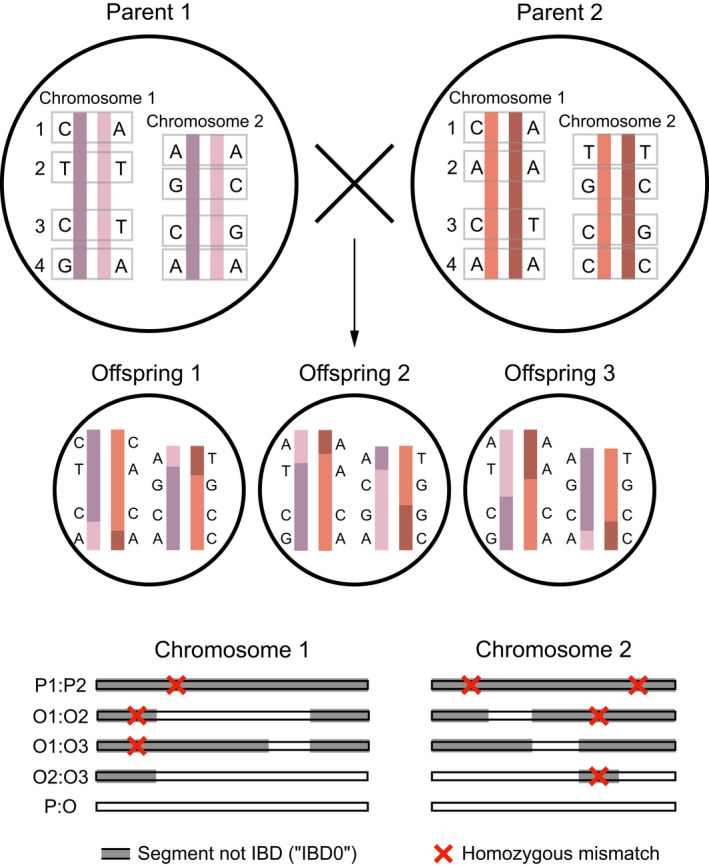
Schematic of metrics used by spore to infer parent‐offspring relationships. Example of two unrelated parents who produce three offspring. Coloured chromosomes denote four different haplotypes (shades of purple and shades of orange), which are recombined and transmitted to three offspring (O1 to O3). Alleles are shown at four loci for each chromatid of two chromosome pairs. Bottom shows a comparison of the fraction of the genome that is not identical by descent (“IBD0,” grey), the variation in IBD0 between chromosomes, and homozygous mismatches (red crosses) across pairs of individuals. Note that full‐sibling (O:O) relationships differ in all three of these values from parent‐offspring relationships (P:O), which are consistently 0: 0% of the genome IBD0, 0 variation in IBD0 (“IBD0 IQR”), 0 homozygous mismatches.

To infer parent‐offspring (PO) relationships from genome‐wide genotypes of a set of individuals, we use three variables (see Figure 2), which we estimate for each pair of individuals: the proportion of the genome that is not IBD (“IBD0”), the variation (interquartile range; IQR) in that proportion among chromosomes (“IBD0 IQR”), and the proportion of loci that carry opposite homozygous genotypes (“homozygous mismatches, HM”). In outbred families, these variables have an expected value of zero in parent‐offspring relationships (and in monozygotic twins), but higher than zero in full siblings and other types of relationships. IBD0 is expected to be invariably zero in all autosomes of parent‐offspring pairs, yielding a total IBD0 of zero. In contrast, in full siblings on average $\frac{1}{4}$ of their autosomes are IBD2, $\frac{1}{2}$ IBD1, and $\frac{1}{4}$ IBD0. However, because of recombination and the independent assortment of chromosomes, this extent of sharing varies between chromosomes, and hence IBD0 IQR will be greater than zero. Notably, interchromosomal variation in IBD0 has not previously been leveraged as a metric to infer genealogical relatives (Huff et al., 2011).

**FIGURE 3:**
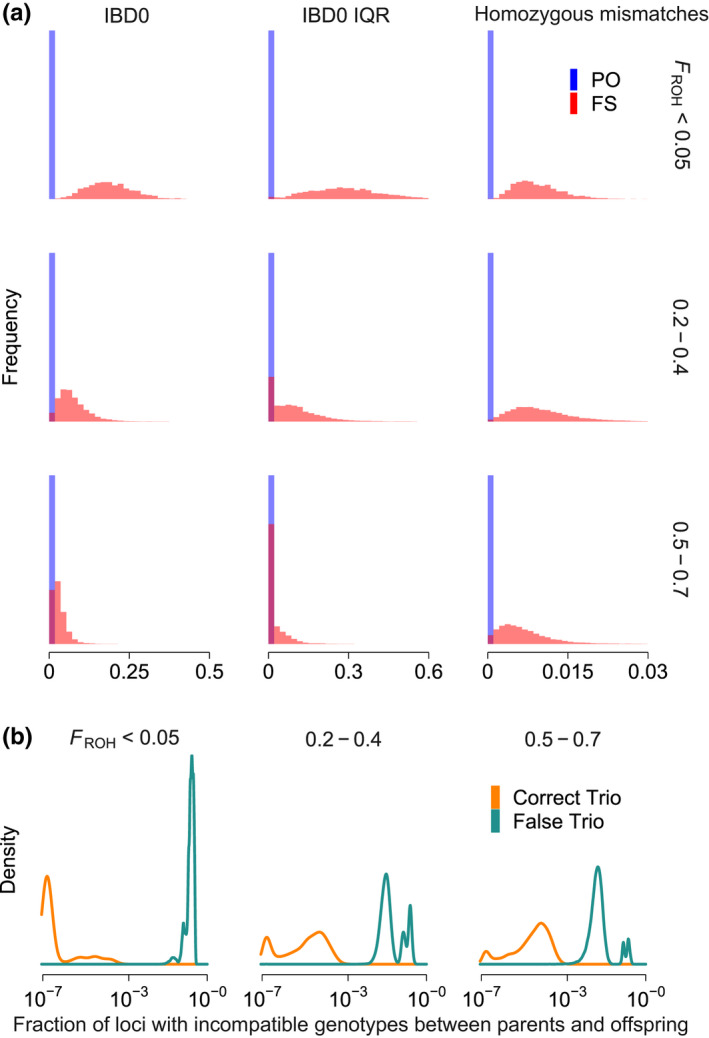
Overview of metrics used by spore to infer parent‐offspring relationships. (a) Example normalized distributions of the variables that spore uses to distinguish parent‐offspring (PO, blue) from full sibling (FS, red) relationships, across levels of inbreeding ($F_{\mathrm{ROH}}$): The fraction of the genome that is not identical by descent (“IBD0”), the interchromosomal variation in IBD0 (measured as interquartile range) “IBD0 IQR,” and the fraction of homozygous mismatches, inferred from 19 simulated autosomes, based on 82,911 PO and 120,291 FS relationships. (b) Example distributions of the fraction of incompatible genotypes (of up to ~2 × 10^6^ loci) in 5909 true trios (orange) and in 111,976 putative, but with at least one incorrect, parents (green) across levels of inbreeding. Data in (a) and (b) are based on the simulated pedigrees with genotyping errors (see Methods S1). Note that the increase in errors of correct trios with increased inbreeding is an artefact of decreased genotype imputation accuracy using low‐coverage sequencing for more inbred samples.

Whereas IBD0, IBD0 IQR, and HM have expected values of zero in outbred parent‐offspring relationships and higher than zero in other types of relationships, these metrics also move towards zero in full‐siblings and other relationships under increased inbreeding (Figure 3a). Furthermore, genotyping errors can result in nonzero values for these metrics in parent‐offspring relationships even in outbred individuals. Hence, by contrast to other methods developed for outbred individuals (e.g., Manichaikul et al., 2010; Qiao et al., 2021), we do not rely on hard‐set thresholds below which we consider relationships to be parent‐offspring. Instead, we use the information in the data set and a researcher‐derived input variable—the assumed average parent‐offspring relationships per individual (APO)—to automatically define those thresholds. Furthermore, we make use of a powerful metric that is also expected to be zero in parent‐offspring relationships: the fraction of loci that carry genotypes that could not have been transmitted from a given set of two parents to their offspring in the absence of germline or embryonic de novo mutations (e.g., an A/A offspring with A/G and G/G putative parents), called “Mendelian trio errors,” to determine correct father–mother‐offspring trios. This metric performs well even under high inbreeding (Figure 3b), but is limited to cases where full trios are present in the sample. Similarly to IBD0, IBD0 IQR, and homozygous mismatches, we also automatically define thresholds below which Mendelian trio errors are considered low enough to be compatible with a true trio. Homozygous mismatches and Mendelian trio errors, like other identity‐by‐state metrics used in alternative genealogical relationship inference methods, are sensitive to SNP ascertainment biases of some genotyping approaches (Waples et al., 2019). However, the additional use of variables derived from called IBD0 segments in spore ameliorates that problem.

We have written spore to be able to incorporate additional user‐provided variables with an expected value of zero in parent‐offspring relationships but higher than zero in other types of relationships. It is also possible to deactivate the use of any or all of the three variables described above. One could, for example, use additional computational resources to calculate R0 (Waples et al., 2019) with ngsrelatev2 (Hanghøj et al., 2019) and incorporate this metric (see Supporting Information “Evaluation of R0 as an alternative parent‐offspring detection metric”) into spore.

### 2.2 Algorithm

**FIGURE 4:**
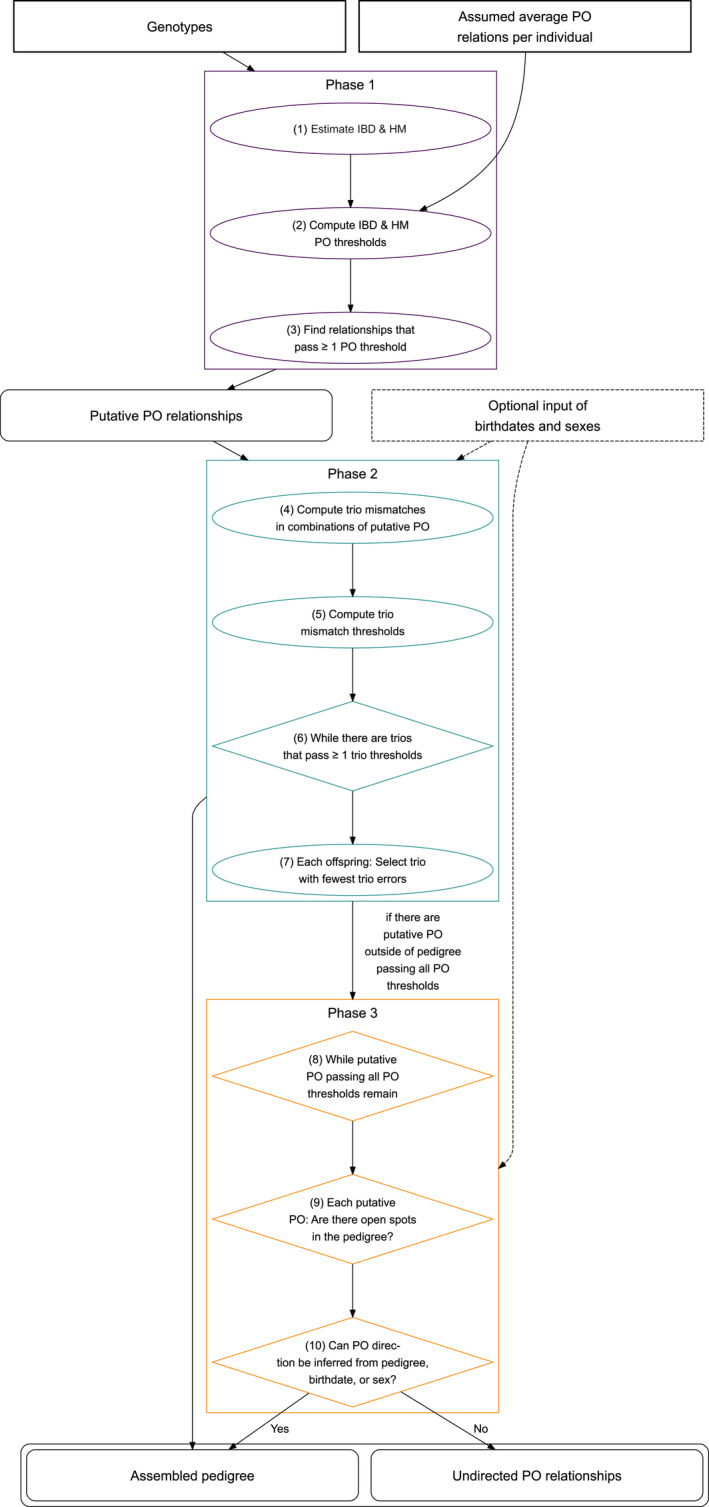
Flowchart overview of the spore algorithm. input is shown as rectangles with square corners, while output is shown as rectangles with rounded corners. Details of each step can be found in the algorithm section.

spore is divided into three phases: (1) Detection of putative parent‐offspring (PO) relationships (Figure 4, steps 1–3), (2) inference of father–mother‐offspring trios within the putative PO relationships (Figure 4, steps 4–7), and (3) inference of PO relationships in the remaining putative PO relationships that could not be assigned to trios (Figure 4, steps 8–10). Details on spore's speed can be found in Figures S1 and S2.

#### 2.2.1 First phase

spore runs truffle 1.38 (Dimitromanolakis et al., 2019) to infer IBD0 between all possible combinations of individuals. Next, spore scans a random subset of the genome (Methods S1 and Figures S3 and S4) to detect homozygous mismatches (opposite genotypes) between individuals, which are divided by the number of loci at which both individuals are genotyped, resulting in a relative measure of homozygous mismatches (“HM”).

Using IBD0, IBD0 IQR, and HM, spore aims to detect putative one‐to‐one PO relationships. To do so, spore uses the only necessary user input (apart from the genotypes themselves), the “assumed average PO relationships per individual” (APO) in the data set (see Performance of varying APO section). This input is used to automatically adjust the thresholds of IBD0, IBD0 IQR, and HM, below which a relationship is declared as putative PO (see Section 2.2.4 below).

#### 2.2.2 Second phase

spore aims to discover father–mother‐offspring trios among the putative PO relationships. This phase can optionally be refined by providing birthdate and sex data. First, spore assembles putative father–mother‐offspring trios out of the putative PO relationships for each focal individual that has at least two such relationships. spore then quantifies the number of loci that contain genotypes that could not have been transmitted from the putative parents to the putative offspring using the mendelian plugin of bcftools version 1.10.2 (Li, 2011). With those Mendelian trio errors, spore once again automatically estimates the thresholds below which a PO trio is considered to be true. If multiple trios pass the threshold(s), the one with fewest errors is assigned as true.

#### 2.2.3 Third phase

spore aims to infer PO relations in the remaining set of putative PO relationships that were so far not detected as a complete father–mother‐offspring trio. Because these relationships can only be detected on a one‐to‐one rather than a more powerful trio basis, spore requires them to pass all three HM, IBD0, IBD0 IQR thresholds (see in Figure S5 how multiple thresholds decrease false positive PO detection). To be placed into the pedigree, these newly inferred putative PO relationships also need to fit into the existing trio‐inferred pedigree. That means that one of the two individuals in a putative PO relationship needs to have unknown parents. If provided, the sex of the unknown parent and the birth order also need to be compatible. While we iterate through one remaining putative relationship at a time, we consider the alternatives for a given parent spot in the pedigree and choose the one with the lowest IBD0, IBD0 IQR, and HM values. Once this is done for all remaining putative PO relationships, spore returns the assembled pedigree. Putative PO relationships that pass all three thresholds but could not be placed into the assembled pedigree, for example because there was no known birthdate or because both directions (parent‐offspring, offspring‐parent) were possible for these two individuals, are output as “undirected PO relationships.”

#### 2.2.4 Thresholding

##### Putative PO relationships

To establish thresholds below which relationships are considered as putative PO, spore calculates the number of individuals that have ≥ 1 PO relationship and > APO PO relationships at levels of IBD0, IBD0 IQR, and HM, separately. This is tested in up to 10,000 increments from the lowest value to the highest, with the first 9000 increments being exactly the 9000 lowest values, and the last 1000 increments being equally sized from the 9000th lowest value to the highest value. We do not exclude high values from this algorithm so spore remains flexible to various levels of genotyping errors at the cost of slightly longer runtime (see 3.3 Cattle population results). Fewer than 10,000 increments are used if fewer than 10,000 unique values exist of the variable that is looked at. The value at which the greatest distance between the fraction of individuals having at least one PO relationship and those that have more than the APO number of PO relationships is then set as the threshold of that variable below which relationships are classified as putative PO (Figure S6A). For example, if spore determined that 100% of individuals would have at least one relationship with IBD0 ≤ 10^−5^ and only 10% of individuals have more than the APO relationships at this threshold, then the distance would be 90 percentage points. If at any other threshold this distance is smaller than 90 percentage points, then 10^−5^ would be chosen as the IBD0 threshold for classifying putative PO relationships in this data set. At this stage, passing one threshold is enough to be classified as a putative PO relationship (see Figure S5 to see how multiple thresholds improve PO detection).

##### Putative father–mother‐offspring trios

To determine the threshold below which Mendelian trio errors are low enough to consider the father–mother‐offspring trio as true, Mendelian errors are used in two ways. In the first case, we aim to quantify how a trio's Mendelian errors compare to other trios that were evaluated for the same offspring. To that end, a trio's Mendelian errors are divided by the mean Mendelian trio errors of the other evaluated trios with the same putative offspring (“offspring‐relative trio errors”). This could be useful if genotype quality varies between individuals, which could increase the errors in both the true and false trios. In the second case, we seek to analyse how low a trio's Mendelian errors are, compared to all evaluated trios (of all putative offspring) across the data set. The resulting variable is simply the percentile of the relative error compared to all relative error counts (“data set‐relative trio errors”).

For both offspring‐ and data set‐relative trio errors, spore then estimates the thresholds below which a trio is considered as true. Since only one set of parents can be true per offspring and only trios comprised of putative PO relationships are tested, spore makes use of two values to estimate the thresholds, (1) the fraction of putative offspring that would have exactly one set of parents, and (2) the fraction of putative offspring that would have more than one set of parents at each threshold. This is tested in up to 10,000 increments from the lowest value to the highest, with the first 9000 increments being exactly the 9000 lowest values, and the last 1000 increments being equally sized from the 9000th lowest value to the highest value. Fewer than 10,000 increments are used if fewer than 10,000 unique values exist of the variable being analysed. The threshold is then set at the trio‐errors value at which the highest fraction of putative offspring are part of exactly one trio that falls below the threshold, but this fraction cannot be lower than the fraction of putative offspring that are part of more than one trio that passes the threshold (Figure S6B). For example, if 90% of putative offspring are part of exactly one trio that is below the fifth percentile of data set‐relative trio errors and there is no threshold with a higher fraction of individuals with exactly two parents, then the fifth percentile is the threshold below which trios are considered to be true.

If only one trio was evaluated for a given putative offspring, only the data set‐relative trio error threshold is used. When more than one trio pass a threshold for a putative offspring, that is, at least two sets of parents seem true, spore chooses the trio that has fewer errors. These cases are also output separately for the user to have the opportunity to examine more closely.

## 3 RESULTS

To evaluate the performance of spore, we applied it to real and to simulated pedigrees of inbred populations. We then compared its performance to king (Manichaikul et al., 2010), crest (Qiao et al., 2021), and sequoia (Huisman, 2017). Unlike spore, these methods are not explicitly designed for inbred populations and can also infer other non‐PO relationships. king provides very fast results in large data sets and is tolerant to population structure, with a major focus on human populations with no inbreeding. crest's advantage is that it can determine more distant relationships, also under the assumption of no inbreeding. sequoia requires only hundreds of SNPs to infer various types of relationships and is robust to some inbreeding. Like spore, crest and sequoia determine the direction (i.e., who is parent, who is offspring) of relationships, while king does not. Table 1 presents an overview of these methods.

**TABLE 1:** Overview of the different algorithms compared with spore and some key differences between them with regards to parent‐offspring inference

Measurement/feature	spore	crest	king	sequoia
IBD	Yes, IBD0	Yes	Yes	No
IBD interchrom. Variation	Yes, IBD0	No	No	No
Kinship	No	No	Yes	Yes
Homozygous mismatches	Yes	No	No	Yes
Mendelian trio comparisons	Yes	No	No	Yes
Automatic thresholds	Yes	No	No	Via likelihood
Infers PO direction	Yes	Yes	No	Yes
Birthdates input	Optional	No	No	Optional
Sex input	Optional	No	No	Optional

We used three data sets to compare the methods: (1) a subset of a population of wild house mice from a long‐term study, (2) five simulated data sets based on the genotypes of the founders of the long‐term house mice study, and (3) a published data set of a cattle pedigree and its genotypes (Alemu et al., 2021; Druet et al., 2020).

### 3.1 Zurich house mouse population

The free‐living wild house mice population has been intensively monitored since 2002, when it was founded using twelve wild‐caught mice from two nearby source populations (König et al., 2015). The mice live in an old barn, which they can freely leave and re‐enter. The barn is regularly searched for new litters and the mice are genotyped at 25 microsatellite loci when they are 13 days old and again as adults (> 17.5 g), and when they are found dead. The genotypes, along with information about the sex and dates of birth and death of individuals (which limits possible fathers to only those alive at the time pups were conceived and, in the case of mothers, when the pups were born) are used for pedigree construction. Only 79% of individuals can be confidently placed in the pedigree using this method (due to incomplete information from microsatellites and death dates), but to test spore we here make use only of individuals that have parents established with high certainty. We analyse a random subset of 151 mice plus their parents—204 mice in total as some of the 151 mice share parents or are parents themselves—of that population, chosen at random among the individuals that we have sequenced (see Methods S1). We imputed the genotypes of the mice using a custom analysis pipeline based on ancestryhmm (Corbett‐Detig & Nielsen, 2017; see Methods S1).

#### 3.1.1 Performance

**FIGURE 5:**
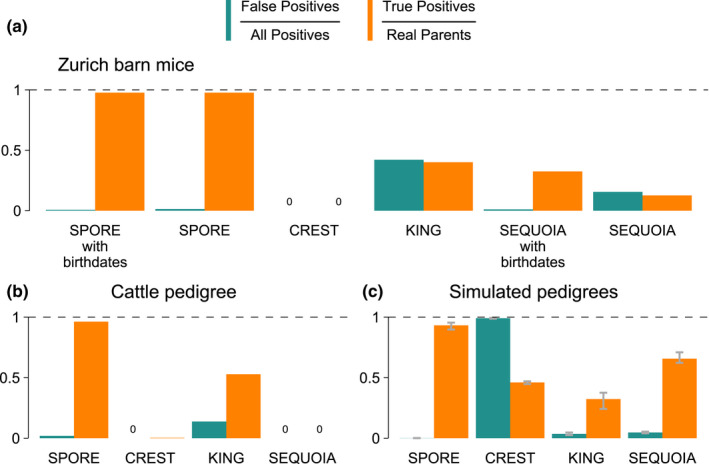
Performance of spore and three other pedigree inference methods. Two real populations are analysed: (a) house mice from the Zurich barn population (mean inbreeding level *F*_ROH_ = 0.3 ± 0.06) and (b) a Holstein cattle pedigree (mean *F*_ROH_ = 0.09 ± 0.03). (c) Performance on five simulated pedigrees (mean *F*_ROH_ = 0.37 ± 0.16). Bar heights represent the mean of each simulation and error bars denote minimum and maximum. spore was run with APO = 6 in extensive‐sampling mode (see Methods S1). spore, crest, and sequoia calls are only evaluated as true if the inferred direction of the call (who is parent, who is offspring) is correct or if the direction was not inferred.

spore detected 295 of the 302 true parents (97.7%) correctly and assigned two parents incorrectly when birthdates were made available and 295 correct with four wrong assignments when birthdates were not used (Figure 5a). crest did not find any parents. king found 40% of parents, but also detected 88 false parents. sequoia performed much better when it was given birthdate information. In that case, it found 32.5% of parents and only detected one wrong parent. Without birthdates, sequoia found only 12.6% of parents while detecting seven wrong parents. Hence, spore found the highest number of correct parents, while making few mistakes.

### 3.2 Simulated genotypes

We simulated five pedigrees (and the 19 autosomes of each individual) of randomly breeding mice of 50 overlapping generations based on the genotypes of the founders (across 2,161,810 variants) of the real house mouse population analysed in the previous section. There were on average 2369.2 (SD = 32.4) individuals per pedigree. Due to the overlapping of generations, offspring were at most at a pedigree depth of 35 equivalents of full generations, which is the unit we refer to in the analysis of specific generations. The pedigrees were simulated without any immigration and hence increasing levels of inbreeding to ~60% of their genome in runs of homozygosity (*F*_ROH_) in the last generation (Figure S7). See Methods S1 for details.

#### 3.2.1 Performance

spore detected 93.2% ± 2.2 (mean ± SD) of the true parents (five simulations with *n* = 4443 ± 70.5 true parents) correctly and detected on average 3.8 ± 1.8 wrong parents (Figure 5c). crest inferred too many pairwise comparisons to be parent‐offspring, so it did find 100% of parents, but only got the correct direction in 46% ± 0.8 of cases. It also found an average of 239,478 ± 67,089 wrong parents. king found only 32.3% ± 5.6 of parents, and also detected 52 ± 9.6 false parents. sequoia found 65.7% ± 3.8 of parents, and 144.2 ± 19.54 wrong parents. Overall, spore made the fewest mistakes, while still detecting more than 90% of true parents.

**FIGURE 6:**
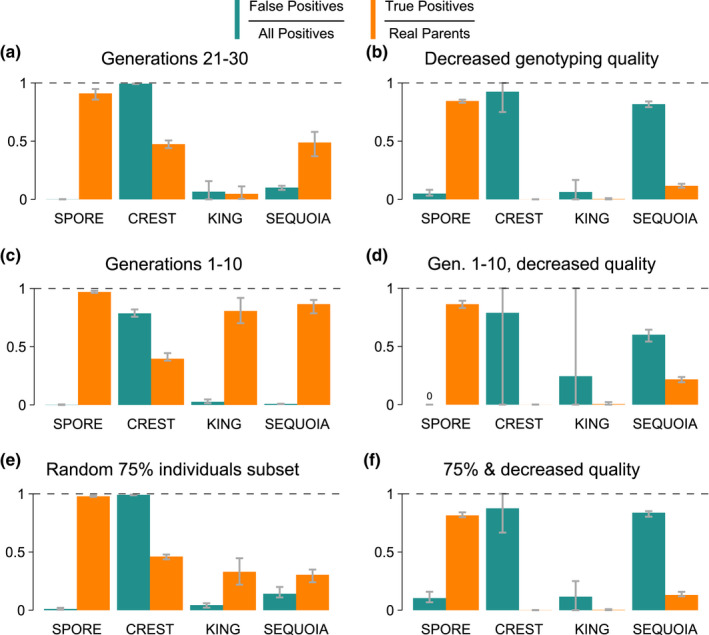
Performance of spore and three other algorithms under increased inbreeding, high genotyping errors and reduced pedigree sampling. Bar heights represent the mean of each simulation and error bars denote minimum and maximum. (a) Same simulated pedigrees as in Figure 5c but only parent‐offspring calls of approximately the last 10 generations are analysed (mean *F*_ROH_ = 0.49 ± 0.07). (b) Same pedigrees as in Figure 5c, but genotype imputation was performed with 10% simulated errors and 0.01× simulated coverage (rather than 2% and 0.03×). (c) the first 10 generations are analysed (mean *F*_ROH_ = 0.17 ± 0.1). (d) a combination of (b) and (c), (e) same pedigrees and genotypes as Figure 5c, but only 75% of individuals were “sampled” (included in the analysis). (f) A combination of b and e. spore was run with APO = 6 in extensive‐sampling mode. spore, crest, and sequoia calls are only evaluated as true if the inferred direction of the call (who is parent, who is offspring) is correct. sequoia false positive calls only decrease by 5.7% (b) 11.4% (d), and 5.6% (f) when direction is ignored.

We also evaluated the impact of increased inbreeding, decreased genotyping quality, and incomplete population sampling through simulated pedigrees. To test performance on inbred individuals, we analysed pedigree generations 21–30 (Figure 6a). These individuals have a considerable level of inbreeding with an average of 49% of their genome in runs of homozygosity (*F*_ROH_ = 0.49 ± 0.07; see Methods S1). spore's PO call accuracy in these generations was very similar to its accuracy over all generations (91% ± 3.4 vs. 93.2% detected true parents with 0.09% ± 0.09 vs. 0.09% of parent‐offspring calls being wrong). crest still detected too many false PO calls (104,496 ± 29,358). king's performance was worse in these late generations than overall, detecting only 4.7% ± 4 versus 32.3% of true parents with 6.64% ± 5.8 versus 3.6% PO calls being wrong. sequoia was the second most reliable algorithm after spore, with 48.8% ± 8.5 parents in generations 21–30 detected versus 65.7% parents in all generations and 10.1% ± 1.7 versus 4.7% false PO calls. Together, the results indicate that spore is superior at inferring PO correctly compared to other methods in controlled simulations with low and also with increasing levels of inbreeding.

With decreased genotyping quality (Figure 6b), but an otherwise unchanged data set, spore still found 84.5% ± 1.2 of true parents with 4.9% ± 2.1 false PO calls. In contrast, crest now only found 0.02% ± 0.05 of the true parents with 92.5% ± 11.2 false PO calls. Similarly, king was reduced to finding 0.3% ± 0.4 of true parents and 6.4% ± 8.8 false PO calls. sequoia found 11.6% ± 1.3 of true parents with 81.8% ± 1.8 false PO calls. Thus, spore is considerably more robust to genotyping errors than other methods.

To disentangle the effects of inbreeding and genotyping error on the inference success of the different methods, we also inferred relationships in the first ten generations (which are the least inbred at *F*_ROH_ = 0.17 ± 0.1) with and without decreased genotyping quality (Figure 6c,d). spore found 97.1% ± 0.7 of true parents without decreased genotyping quality and 86.3% ± 2.4 with decreased genotyping quality, whereas 0.1% ± 0.2 and 0% ± 0 of PO calls were wrong, respectively. In contrast, crest's results changed from detecting too many PO relationships (78.6% ± 2.5 false PO calls) without decreased genotyping quality to detecting almost no true parents (0.03% ± 0.07 of true parents) with decreased genotyping quality. On the other hand, king detected 80.7% ± 8.4 and 0.5% ± 1 of true parents with 2.7% ± 1.3 and 24.4% ± 43.3 wrong PO calls, respectively, showing a marked difference in performance between data sets with varying genotyping quality. Similarly, sequoia detected 86.6% ± 4.7 of true parents with 0.9% ± 0.2 false PO calls without decreased genotyping quality, but only 21.7% ± 1.8 of true parents with 60.1% ± 4.2 false PO calls with decreased genotyping quality. These results are qualitatively similar to results based on individuals with minimal inbreeding (*F*_ROH_ ≤ 0.05; Figure S8). In summary, spore is more robust to genotyping errors even under minimized inbreeding.

Sampling a random 75% of individuals in the data set (*n* = 2504 ± 83.1 true parents) and hence no longer including the entire simulated pedigree, also affected the quality of inference (Figure 6e). spore, which works best the more full trios are in a data set, still found 97.9% ± 0.5 of true parents, but now also 1.1% ± 0.7 of PO calls were incorrect. crest again detected too many false parents (136,718 ± 36,558). king found 33% ± 9 of true parents, but also 4.4% ± 1.4 false PO calls. sequoia found 30.4% ± 4 of true parents and also 14.2% ± 3.5 false PO calls. When sampling is even less complete (50%), spore still detected 93.1% ± 3.1 of PO relationships but false positives also increased to 21.9% ± 8.5 (Figure S9). An alternative “intermediate sampling mode” allows spore to detect 86.6% ± 4.8 of PO relationships with only 0.2% ± 0.1 false PO calls in this scenario (see Figures S10–S12 for performance under varying sampling densities). crest called essentially each pair as PO, while king and sequoia have a smaller increase in false positive calls than spore. Combining decreased genotyping quality with a 75% subset (Figure 6f) further decreased inference quality. Nevertheless, spore still found 81.4% ± 1.8 of true parents with 10.3% ± 3.6 false PO calls. By comparison, crest only found 0.04% ± 0.06 of true parents with 70% ± 41.5 false PO calls. king detected only 0.3% ± 0.34 of true parents, with 11.6% ± 11.2 false PO calls. sequoia detected 13.1% ± 1.6 of true parents and 83.7% ± 2 wrong PO calls. Altogether, the results indicate that spore is a more accurate method when analysing inbred, incompletely sampled pedigrees even when there are abundant genotyping errors.

##### Performance with varying APO

**FIGURE 7:**
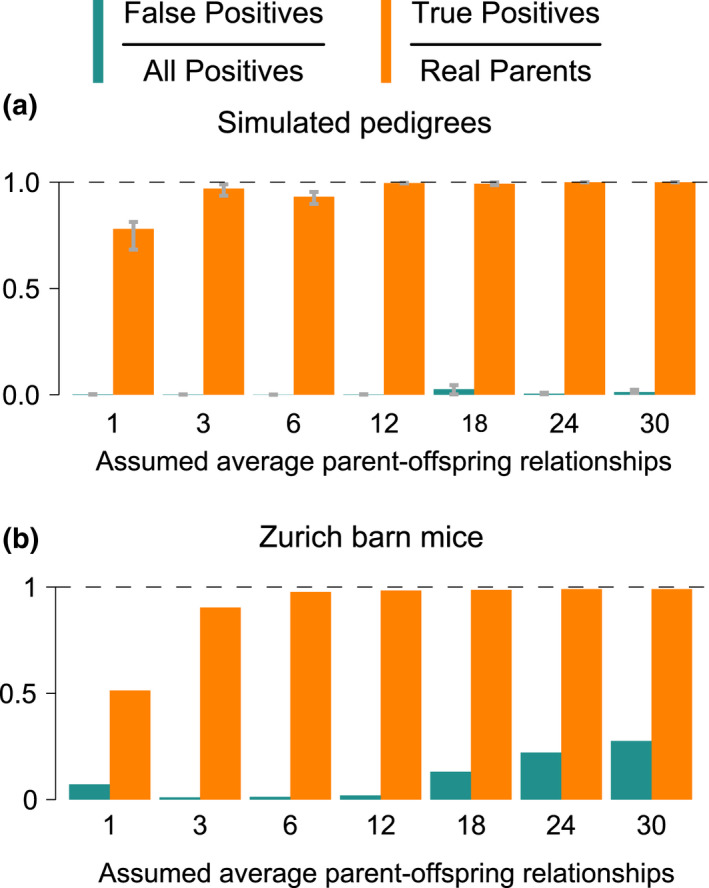
spore with different assumed average parent‐offspring relationships per individual. spore was run in extensive‐sampling mode. Six is the setting used for spore in all other plots. (a) Bar heights represent the mean of the five simulations and error bars denote minimum and maximum. (b) Results for the house mouse long‐term study data set.

To evaluate the impact of the user input “assumed parent‐offspring relations per individual” (APO), we set it to a range from the lowest possible (1) to quintuple (30) of the value we used otherwise (6). Overall, spore detected more true parents at higher APO, but also slightly increased the false positives (Figure 7a). In the simulated pedigrees, the entire range of APO values delivered similar results, with at least 78 ± 5.5% detected true parents and at most 2.6 ± 2.1% false PO calls. However, APO had a greater impact on the quality of the inference in the house mouse study population: intermediate APO values led to similar results, while extreme values led to many missed or falsely inferred parents (Figure 7b). In sum, spore is not particularly sensitive to a range of APO but will have diminished accuracy at extreme values.

spore's APO‐based thresholding is also robust to reproductive skew between the sexes, with APO best set at the assumed average PO relations overall, independent of sex, or a low value in general (see Supporting Information section APO under reproductive skew).

### 3.3 Cattle population

To test spore's performance in an independent real pedigree, we applied it to a published set of 150 cattle offspring and their 100 unique parents and some grandparents, resulting in an incomplete pedigree with 320 known PO relationships (Alemu et al., 2021; Druet et al., 2020). We found high IBD0 values between parents and offspring (mean = 0.5; SD = 0.17; expected mean and SD = 0), suggesting a high genotyping error rate that would allow us to test spore's performance under these conditions.

#### 3.3.1 Performance

spore detected 96.3% of the true parents (*n* = 320) correctly and detected six wrong parents (Figure 5b). crest found 0.3% of parents, with 0 false parents. king found 52.8% of parents, but also detected 27 false parents. sequoia did not find any parents. Thus, spore is superior at detecting parents in this independent real pedigree with high genotyping error rate.

## 4 DISCUSSION

Our results based on data from a real house mouse population, a real cattle pedigree, and simulated pedigrees, show that spore infers parent‐offspring relationships in data sets with inbreeding or with high genotyping error, or both, with considerably more accuracy than other methods. The high performance of spore is based on (1) its reliance on an integrated combination of robust metrics, such as identity‐by‐descent of chromosomal segments, incompatible genotypes within trios and within parent‐offspring pairs; (2) on the focus on parameters that have fixed expected values in parent‐offspring relationships regardless of inbreeding levels; and (3) on the automatic adjustment of thresholds to be flexible to genotyping errors, which is a key difference to many other approaches.

Another advantage of spore is that it has few requirements. It does not need a genetic map and can optionally leverage sex information and birthdates. The minimal input—a VCF and a simple config file—also facilitates use by less bioinformatics‐experienced investigators. More advanced use is also supported by the ability to incorporate additional metrics with fixed expected values in parent‐offspring pairs, but not other relationships.

A caveat of spore is that it works on the assumption that there are parent‐offspring trios in the analysed data set. When ~15% or more of individuals are part of a trio, false positive calls achieve an asymptotic low value < 1% (Figure S12). This assumption, however, is likely met in many of spore's intended uses. Furthermore, spore is designed to identify only parent‐offspring relationships. However, parent‐offspring inferences can then be used to identify siblings (individuals sharing parents) and to assemble multigenerational pedigrees. Pedigrees could be further completed using other methods such as sequoia's full‐sibling inference, although we have not evaluated its performance under inbreeding or imperfect genotypes.

We suggest that spore will be a useful tool for researchers monitoring experimental (e.g., Ferrari et al., 2022; Frentiu & Chenoweth, 2008; Luzynski et al., 2021; Stockley et al., 2013), wild (e.g., Bonnet et al., 2019; Chen et al., 2019; Clutton‐Brock & Pemberton, 2003; Weinman et al., 2015), agricultural (e.g. McClure et al., 2018; Tortereau et al., 2017), and even some human (e.g., Arciero et al., 2021), populations with incomplete mating and birth tracking, potentially imperfect genotypes, and at least some autozygosity resulting from population history or recent mating between close relatives.

## AUTHOR CONTRIBUTIONS

Conceptualization: Jan‐Niklas Runge, Anna Lindholm, Barbara König and Andres Bendesky. Investigation: Jan‐Niklas Runge and Anna Lindholm. Formal analysis: Jan‐Niklas Runge. Methodology: Jan‐Niklas Runge and Andres Bendesky. Resources: Anna K. Lindholm and Barbara König. Software: Jan‐Niklas Runge. Visualization: Jan‐ Niklas Runge. Supervision: Andres Bendesky. Funding Acquisition: Jan‐Niklas Runge, Anna K. Lindholm, Barbara König and Andres Bendesky. Writing: Jan‐Niklas Runge, Anna K. Lindholm, Barbara König and Andres Bendesky.

## CONFLICT OF INTEREST

The authors declare no conflicts of interest.

## FUNDING INFORMATION

Swiss National Science Foundation grants P1ZHP3_181303 & P2ZHP3_195249 (JNR), 31003A_176114 (BK), 31003A_120444 & 31003A_160328 (AL). Searle Scholarship, Klingenstein‐Simons Fellowship, Sloan Foundation Fellowship, and National Institutes of Health award R35GM143051 (AB).

## Supporting information


Appendix S1

## Data Availability

Sequence reads of the house mice from the long‐term study have been made available in NCBI Sequence Read Archive under BioProject PRJNA782421. Founder genotypes used for imputation and simulation are available in Zenodo under doi 10.5281/zenodo.6465088. The cattle pedigree genotypes are available in Dryad under doi 10.5061/dryad.vx0k6djq8 (Druet et al., 2020). The spore version used here is archived in Zenodo under doi 10.5281/zenodo.6465798. spore is available at https://github.com/jnrunge/spore.
