# Supplementary material for: Parent‐offspring inference in inbred populations
Source: Mol Ecol Resour. 2022 Jul 22;22(8):2981–93. doi: 10.1111/1755-0998.13680 (PMC9796703; doi:10.1111/1755-0998.13680)
Supplement: Supplementary file 1 — Appendix S1 [file MEN-22-2981-s001.pdf]

Supporting Information for

'Parent-offspring inference in inbred populations'

Jan-Niklas Runge, Barbara König, Anna K. Lindholm & Andres Bendesky

Supplemental Methods

Genomic inbreeding coefficient calculation

To calculate inbreeding coefficients in house mice, in simulated genomes, and in cattle, we used the fraction of the genomes that are part of continuous runs of homozygosity (F_{ROH}), an empirical measure of inbreeding. We used a cutoff of ≥ 1 Mb ROH (Szpiech et al., 2013). Runs of homozygosity were identified using *bcftools roh* function (Narasimhan et al., 2016), using allele frequencies of the analyzed populations. We used a fixed likelihood of 30 for unseen genotypes (the recommended setting when genotyping likelihoods are absent) to account for genotyping errors and a 10^{-8} M / bp genetic map, a good approximation for mammals (Dumont & Payseur, 2008). The summed length of ROH ≥ 1 Mb on autosomes was divided by the reference length of those chromosomes, resulting in the fraction F_{ROH}.

Values of F_{ROH} for other populations were extracted from the literature: Devils Hole pupfish (called using *bcftools* 1.10.2 (Li, 2011), 1 Mb cutoff, extracted from plots in Tian et al. (2021) using *WebPlotDigitizer* (Rohatgi, 2021)), industry/wild chicken (called using *PLINK* with a 300 kb cutoff by Talebi et al. (2020)), bottlenecked wolf (ROH identified using likelihood ratio estimation, cutoff of ≤ 10 generations recent ancestry, inferred from genetic map by Kardos et al. (2018)), Sao Paolo Quilombo (ROH detected with *PLINK*, 500 kb cutoff, by Lemes et al. (2018)), and UK Biobank (called with *PLINK*, no cutoff, by Johnson et al. (2018)).

Figure generation of figures 1 & 3 & S5

F_{ROH} values in **Main Figure 1 A** were extracted as described in the section *Genomic inbreeding coefficient calculation*. **Main Figures 1 B & 3 A** show IBD values extracted from *TRUFFLE* and homozygous mismatches extracted from *SPORE*, which were run with the simulation imputed genotypes (with few genotyping errors, the same as in **Main Figure 5 C**). F_{ROH} values were calculated as described in *Genomic inbreeding coefficient calculation*, based on imputed genotypes. The parent-offspring and full-sibling groups are based on the 'true' simulated pedigree.

Main Figure 3 B shows Mendelian trio mismatches extracted from *SPORE* (calculated using *bcftools*) run on the same simulations. This means that the true and false trios are the ones that *SPORE* evaluated, hence only trios that are made up of putative parent-offspring relationships—having passed at least one of *SPORE's* three thresholds (IBD0, IBD0 IQR, homozygous mismatches)—are evaluated. Since this data is based on imputed genotypes, mismatches in true trios increase with inbreeding due to imputation working slightly less well with increased inbreeding. Nonetheless, errors were orders of magnitudes lower in true trios. **Figure S5** shows how many true and false PO pass *SPORE*-generated thresholds for the three variables IBD0, IBD0 IQR, and homozygous mismatches based on the simulated pedigree genotypes, using APO = 6 as a setting for *SPORE*.

SPORE (Specific Parent-Offspring Relationship Estimation)

SPORE is written in *R* 3.6.3 (R Core Team, 2018) with the packages *data.table* 1.13.2 (Dowle & Srinivasan, 2019), *dplyr* 1.0.2 (Wickham et al., 2019), *ggplot2* 3.3.2 (Wickham, 2016), *MASS* 7.3.53 (Venables & Ripley, 2002), *naturalsort* 0.1.3 (Abe, 2016), *readr* 1.4.0 (Wickham et al., 2018), *stringr* 1.4.0 (Wickham, 2019), *sys* 3.4 (Ooms & Csárdi, 2020), *tidyr* 1.1.2 (Wickham & RStudio, 2021). In addition to these packages, we also use *optiSel* 2.0.3 (Wellmann, 2021) for the analyses presented here.

Speed

SPORE runs for 8 hours on one core for a pedigree of 2500 individuals. However, *SPORE* can take advantage of multiple cores in some key parts of its algorithm, approximately halving its run time on four cores compared to one and *SPORE* can also optionally skip steps when rerunning it on the same data. Details on the speed of *SPORE* can be found in **Figures S1 & S2**.

IBD calculation

We used *TRUFFLE* 1.38 (Dimitromanolakis et al., 2019) to calculate IBD0 values for each relationship. We excluded variants with a minor allele frequency `-maf` below 10^{-4} and/or >95% missing genotypes at that locus, the default *SPORE* values, which will not filter out low-frequency variants in our datasets, since they are smaller than 10,000. Both settings

can be customized in the *SPORE* options file. To extract IBD0 values for all chromosomes separately, we also ran *TRUFFLE* on VCF files containing only one chromosome at a time. This is automatically done within *SPORE* for all chromosomes found in the input VCF file. IBD0 IQR was calculated as the interquartile range — which is less sensitive to outliers than the variance — of all IBD0 values of each of the autosomes.

Homozygous mismatches

We calculate homozygous mismatches on a user-specified random fraction of the genome to decrease run time. The *SPORE* default and value used in this manuscript is 0.01. The impact of other values on run times and results are shown in **Figures S2 to S4**. We then run a custom *R* script that counts the loci with homozygous mismatches for every pair of individuals and the loci where both individuals compared are genotyped. We then calculate the fraction of loci genotyped in both individuals that have homozygous mismatches for each pair.

Mendelian trio mismatches

We calculate genotype mismatches in possible father-mother-offspring trios using the *mendelian* plugin of *bcftools*. Trios are deemed possible and thus worthy of this time-intensive computation in all cases where a given individual (the putative offspring) 1) has a putative PO relationship to both putative parents, 2) optionally available birthdates are not older for the offspring than for the parent, and 3) optionally available sexes differ between the two parents. All trios fulfilling these criteria are then passed on to *bcftools* for the computation of the mismatches. We extract a relative measure of these mismatches by dividing the number of loci with mismatches by the number of loci that were genotyped in all three individuals in a putative trio. A mismatch can be a heterozygous offspring genotype when both parents are homozygous for the same allele or a homozygous offspring genotype when each parent is homozygous for opposite alleles.

Setting for samples with few trios

Because *SPORE's* performance by default in "extensive sampling mode" benefits strongly from dense sampling of pedigrees, we added an optional mode that modifies how *SPORE* detects thresholds for Mendelian trio errors. This mode does not change the fundamental principle of *SPORE* where we make use of a prior knowledge of there being at least some parent-offspring trios in the sample. The only change here is that thresholds for Mendelian trio errors will be set lower, resulting in fewer trios being evaluated as true.

In detail, the Mendelian trio error thresholds are not chosen to be those values with the highest proportion of putative offspring being in precisely one trio that passes the threshold. Instead, the first peak in the fraction of putative offspring having exactly one trio is chosen. We chose this approach because we observed that this fraction was remaining around similar values for a large number of tested thresholds, likely because more and more false trios

passed the increasing thresholds, balancing out the decrease in individuals with a true trio increasingly having more than one trios passing the threshold, thus choosing higher thresholds than intended.

We demonstrate the performance of this alternative setting in even more ‘downsampled’ inbred pedigrees (the downsampling means that x % of the individuals in the pedigree were chosen at random to remain in the dataset). Here, combined with APO=1, *SPORE* finds at least 80% of true parents in standard genotyping quality, but the fraction of PO calls that is wrong depends on how extreme the subset is: as high as 73% in one 10% sampled population, but down to 10.9% $\pm$ 0.07 wrong PO calls at 20% sampling (**Figure S10**). Under decreased genotyping quality, *SPORE* returns numerous false positive parent-offspring relationships.

For reference, the 50% downsampling results in an average of 74% of individuals having a PO relationship, and 24% of individuals having a trio relationship in the dataset. At 25%, these numbers are 41% and 5.8%, at 20%, they are 35% and 3.7%, at 15% they are 26% and 2%, and at 10% they are 18% and 1% (**Figure S11**). We sub-sampled each of the original five pedigrees once for the 50% condition, and 11 times for the other conditions.

To assess the required fractions of the individuals that need to have a full trio relationship for *SPORE* to deliver acceptable results, we built a linear mixed model with the natural logarithm of the false positive rate of *SPORE* as the dependent variable and the fraction of IDs with full trios as the independent variable, with the simulation repetition (n=5) as a random effect (**Figure S12**). This model is based on the sampling subsets between 10 and 50%, as the aim was to approximate the fraction of samples with full trios necessary for desired false positive rates. Based on model predictions and 95% confidence intervals (generated using *merTools*'s *predictInterval* (Knowles & Frederick, 2020) with 1,000 simulations), we find that for a false positive rate below 1%, between at least 12% and 17% of the population will need to be in full trios (median to upper 95% CI). For a false positive rate below 5%, one needs 7% to 11%, and for a false positive rate below 10% one needs 4% to 9%. Note that these numbers are based on considerable inbreeding and are expected to decrease with more moderate inbreeding levels.

Datasets

Zurich house mouse population

Sequencing We sequenced the twelve founders (F_0) of the population and eight of their F_1 offspring (at least one for each founder) to an average of 9.2x (SD=1.16) coverage after removing duplicates. We used the Illumina HiSeq X platform with paired-end reads of 150 base pairs. We aligned all reads to the reference *Mus musculus* genome (GRCm38.p6) using the *mem* algorithm of *bwa* 0.7.17-r1188 (Li, 2013), sorted the resulting bam files and marked duplicates using Picard toolkit 2.18.26 (Broad Institute, 2019). We called genotypes using *bcftools* 1.10.2 (Li, 2011) and *Strelka* 2.9.10-0 (Kim et al., 2018), both filtered independently to only include autosomal bi-allelic loci with a minimum variant quality (‘QUAL’) of 100, no missing genotypes, $\geq$ 3 reads per genotype, but otherwise no genotype quality filtering at

this stage. We then used the *isec* function of *bcftools* to extract those genotypes that were supported by both genotype callers. Next, we removed loci with genotypes that were found to disagree within the F_0-F_0-F_1 trios (Mendelian errors) using *vcftools* 0.1.16 (Danecek et al., 2011). Finally, we removed loci that were found to be in regions of particularly high (> 25x) or low (< 5x) average coverage across the F_0F_1. We phased those genotypes using the known pedigree with *WhatsHap* 0.18 (Martin et al., 2016) based on the sex-averaged genetic map published by Liu et al. (2014). Afterwards, we only kept loci in which all genotypes had at least a genotype quality score of 20. In the end, we detected 2,161,810 loci with variation among the founders.

We chose 204 individuals (making up the 302 unique parent calls) from later generations, F_x. The mice were sampled between 2007 and 2011, and 5,803 mice had been sampled up until the last mouse analyzed here was sampled. We then sequenced these individuals on the Illumina NextSeq 500 platform with single-end 75 base-pair reads and alternatively on the Illumina NovaSeq platform with paired-end 150 base pair reads to an average 0.03x (SD=0.01) effective coverage (the fraction of loci of interest with at least one read, see J. H. Li et al., 2021). We aligned all reads to GRCm38.p6 using the *mem* algorithm of *bwa* and marked duplicates using Picard. From these aligned reads, we extracted positions at the 2,161,810 loci with variation in the founders with a base quality ≥ 30 using the *mpileup* function of *samtools* 1.10 (Li et al., 2009). For each individual, we excluded variants within 300 bases of another variant to reduce bias towards one strand due to several bases being in one read pair. These bases were then used as input for genotype imputation.

Genotyping via imputation To genotype the house mice in the long-term study, we designed an imputation pipeline that converts the output of the ancestry inference software *AncestryHMM* 0.94 (Corbett-Detig & Nielsen, 2017) into genotypes. In short, the pipeline takes phased genotypes of the twelve founding mice of the population and uses these as 24 ancestral genomes. From these ancestral genomes and the low-coverage data of each of the 204 individuals that we analyzed, *AncestryHMM* infers probabilities for each ancestry combination at each locus. Each of the 24 ancestral genomes was assumed as having equal representation in the overall population, and for each individual the foundation of the population was assumed to be 10 generations in the past, but this was not fixed, i.e. *AncestryHMM* could deviate from it. We assumed a genotype error rate of 0.1% and used a genetic map with 10^{-8} M / bp.

AncestryHMM outputs the probability of each ancestry combination (e.g. ancestry 1 and 3) at each locus. To convert this into genotypes, we first interpolated the probabilities at loci in between the ones where bases were available. Next, we summarized the probabilities for each ancestry by taking the probability for this locus to be homozygous for ancestry y and adding half of the sum of all probabilities for this locus to be heterozygous for ancestry y, because the probability to be heterozygous represents the probability for only one chromosome (per locus) to descend from this ancestry.

$$\sum P_y = P_{y_{\text{Hom}}} + 0.5 \cdot \sum P_{y_{\text{Het}}}$$

We multiplied this total probability P_y for ancestry y at each locus with the allele of this ancestry (reference allele = 0.0, unphased = 0.5, and alternative allele = 1.0). We did this for all 24 ancestries and then summed these allele values up at each locus to receive an imputed proportion of the alternative allele for each locus of each offspring (between 0 and 1). Imputed proportions between 0.0 and 0.01 were inferred as a homozygous reference allele genotype, between 0.495 and 0.505 as heterozygous, and between 0.99 and 1.0 as homozygous alternative allele. This means that 99% of the *AncestryHMM*-inferred ancestries agreed on the genotype.

For the long-term study population of house mice, we removed genotypes in the first and last 10 Mb of each chromosome for the analyses here, because we detected a slightly increased genotype error rate in those regions.

Simulated pedigrees

We simulated genotypes of mice for 50 time intervals (which are roughly equivalent to generations that can overlap) using *SimuPOP* 1.1.10.9 (Peng & Kimmel, 2005) based on the genotypes of the 12 mice that founded the long-term study population (2,161,810 loci as above), the same that were used as ancestral alleles for imputation. This resulted in an average of 2,369.2 (SD=32.4) individuals per pedigree. Genotypes were simulated at their respective loci, i.e. loci kept the same position as implied by the reference genome (GRCm38.p6). The genetic distance in Morgans between loci, and thus the probability of recombination, was based on the sex-averaged map published by Liu et al. (2014).

We simulated overlapping generations in which each individual lives for two time intervals after their birth and could mate in both of those. Individuals were simulated to mate randomly, but could only produce Pois_{50} offspring each generation, and each randomly mated pair would produce Pois_{6} offspring, with Pois_{λ} being a Poisson distribution. Hence, by chance, some individuals could have multiple litters with Pois_{6} offspring, but many would also have no offspring in a generation. This also means that there were about 150 individuals alive and available as mates at any given moment. Litter size and offspring per time interval are within the typical ranges observed also in the long-term study (Ferrari et al., 2019; König & Lindholm, 2012). We simulated five unique pedigrees with these settings.

To simulate erroneous and low-coverage sequencing, we extracted bases from all the simulated genotypes of each simulated individual with a probability of Pois_{cov}, with "cov" being the coverage (0.03, with the exception of 0.01 in "decreased genotyping quality" analyses). Furthermore, each extracted base was simulated to be 'misread' as the other possible base from its bi-allelic locus with a probability of Pois_{err}, with "err" being 0.02 or 0.1, with 0.02 being used in the main analyses and 0.1 in the "decreased genotyping quality" analyses.

The simulated reads were then used as the basis for the imputation pipeline described above. Hence, all analyses of simulated mouse genotypes presented here are based on imputed genotypes, which contain some errors due to either limits of the imputation in general or the base errors that we deliberately introduced. This is done to create a dataset that is comparable to the genotypes of the real house mice we also analyzed.

In the end, the simulated pedigrees cover a wide range of inbreeding levels, measured through F_{ROH} (**Figure S7**). Any mention of the generation of an individual is based on the 'true' pedigrees of the simulations and the summary statistic "equiGen" of the *R* package *optiSel*, which represents the "number of equivalent complete generations," i.e. is adjusted for matings within generations. When only specific generations were analyzed, the output files of the methods and the true pedigree were subset to only include relationships and trios within individuals from the targeted generations, e.g. if one parent was not of the targeted generations, none of their children would be included in the analyses.

Cattle

We converted the published set of 13,037,955 loci (derived from whole-genome short-read Illumina sequencing using Illumina HiSeq 2000 by Druet et al., 2020) into VCF format. We filtered out loci with more than 10% missing genotypes and loci without allelic variation, and only kept the autosomes for the analyses, yielding a final set of genotypes at 12,653,341 loci.

Execution of other algorithms

CREST 1.0.0 was run on IBD data inferred by *IBIS* 1.20.9 (Seidman et al., 2020), as recommended by *CREST* developers (Qiao et al., 2021). *IBIS* was run using the genetic map of the Collaborative Cross from Liu et al. (2014) for *Mus musculus* and simulations and the genetic map from Ma et al. (2015) for cattle.

KING 2.2.5 had to be used with pruned genotypes (using *PLINK*) as it would otherwise not complete. Parameters were chosen to prune as little as necessary for execution to succeed. In the end, we chose 200 kb windows that have a correlation of $R^2 < 0.3$ for the long-term study, $R^2 < 0.5$ in 250 kb windows for the simulations, and $R^2 < 0.75$ in 10 kb windows for cattle. There was one exception in the simulations that required pruning in 300 kb windows in the "decreased genotyping quality" condition for *KING* to successfully complete.

SEQUOIA 1.3.3 was run without sibling inference and both pedigree parent-offspring relationships and potential parent-offspring relationships (`GetMaybeRel` function) were considered, though the latter gave only few additional results. We pruned the genotype data because *SEQUOIA* is designed to run on genotypes in the hundreds. We used $R^2 < 0.1$ in 1 Mb windows for the long-term study population of house mice and simulated genotypes, with the exception of "decreased genotyping quality" conditions, which required pruning to $R^2 < 0.01$ in 5 Mb windows to allow *SEQUOIA* to finish running (within 5 days). We used $R^2 < 0.01$ in 10 Mb windows for the cattle due to the larger number of loci in the unpruned dataset (ten times more than in the house mouse datasets).

Sex information was provided to *SPORE* and *SEQUOIA* in all runs, while birthdates were only made available to both in the long-term mouse data.

We considered additional methods but did not analyze their performance for the following reasons. We did not further analyze *CLAPPER* (Ko & Nielsen, 2017), because it is limited

to inferring relationships of individuals from five generations at a time. *PRIMUS* (Staples et al., 2014) was excluded because it did not run successfully with the IBD values in real and simulated mice analyzed here. We also did not use *AlphaAssign* (Whalen et al., 2019) because it was designed primarily for cases where there is some knowledge of possible parents and where close inbreeding is not likely. Finally, *BONSAI* (Jewett et al., 2021) was also not evaluated, because it is intended for sparse pedigrees and contains hard-coded IBD and age distributions optimized for humans.

APO under reproductive skew

The key input variable of *SPORE*, the number of assumed parent-offspring relationships per individual, is expected to approximate the average number of parent-offspring relations per individual, independent of skew. For simulated pedigrees, we allowed for differences in the parent-offspring relations to occur, but did not explicitly test *SPORE's* robustness to an extreme skew of reproductive success, as can occur between sexes or within sexes in many species. To test this directly, we created a highly skewed and incomplete pedigree with 1,286 individuals where one sex contributed 624 parents and the other only 8, with 1,226 detectable parent-offspring relationships. Instead of simulating this pedigree, we sampled IBD0 values from the already simulated data and gave each relationship a random, simulated IBD0 value, depending on whether it was parent-offspring or not (hence, including the effects of inbreeding and some genotyping errors). We then ran the *SPORE* thresholding algorithm on this IBD0 data with four APO values that researchers could end up using: 1 (the lowest possible and roughly the average for one sex), 2 (the closest to the mean number of offspring per individual of 1.9), 90 (the highest number offspring in the pedigree for an individual), and 45 (the midpoint between the extreme ends of the distribution). We found that the best performance was achieved by using an APO of 2, closest to the true mean of the sample. With this, 95.8% of true parent-offspring (PO) relations were below the resulting threshold, and only 2.6% of relationships below the threshold were not PO. At APO=1, 74.4% of true PO were found, with only 0.44% of below-threshold relations not being PO. In contrast, APO=45 and APO=90 allowed all true PO relationships below threshold, but also many non-PO relationships (93.3% and 75.3% non-PO passed the threshold, respectively).

Evaluation of *R0* as an alternative parent-offspring detection metric

In addition to IBD0, IBD0 IQR, and homozygous mismatches, *R0* (Waples et al., 2019) is also expected to be zero in parent-offspring (PO), but not other relationships. We evaluated the performance of inferring parent-offspring relations based on *R0* being zero, calculated using *ngsRelateV2* (Hanghøj et al., 2019) from pruned, as recommended, data using a cutoff of $R^2 < 0.1$ with 100kb sized windows. While *R0* performs remarkably well for a single-measure based approach under high genotyping quality, with 90.6% $\pm$ 0.4 true PO relations with *R0* = 0 (*SPORE*: 93.2%) and only 0.8% $\pm$ 0.4 (*SPORE*: 0.09%) of relations with *R0* = 0 not being PO. However, the measure is quite sensitive to genotyping quality, with still 85.6% $\pm$ 1% (*SPORE*: 84.5%) of true PO at *R0* = 0, but 31.6% (*SPORE*: 5%) of relations with *R0* =

0 not PO under decreased genotyping quality. In the Zurich house mouse population, only 32.8% (*SPORE*: 97.7%) of true PO relationships have $R0 = 0$, and 5.7% (*SPORE*: 1.3%) of relationships with $R0 = 0$ were not PO. In the cattle pedigree, 32.8% (*SPORE*: 96.3%) of PO relations were $R0 = 0$ and 0 (*SPORE*: 1.9%) of relations with $R0 = 0$ were not PO.

Supplemental Figures

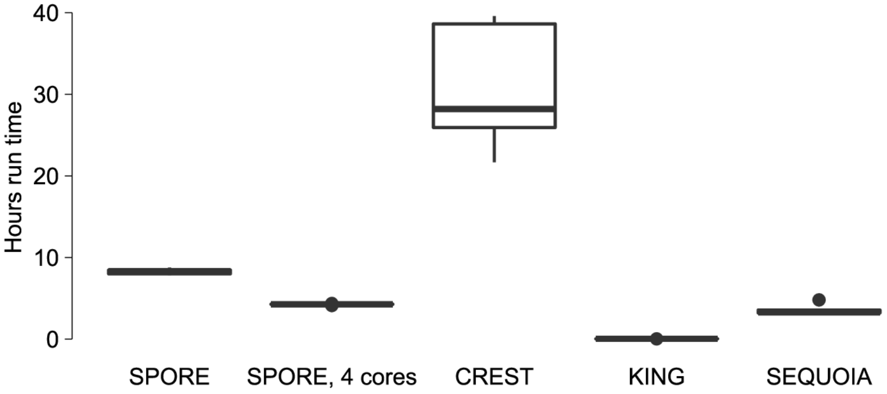

Figure S1: Runtimes in the simulated data sets for the four algorithms. Boxes denote the interquartile range (IQR) with the line at the median and whiskers extend to 1.5 times the IQR. For a practical comparison, these times include IBD detection (*TRUFFLE* for *SPORE* and *IBIS*, as recommended, for *CREST*) and pruning (*KING*, *SEQUOIA*).

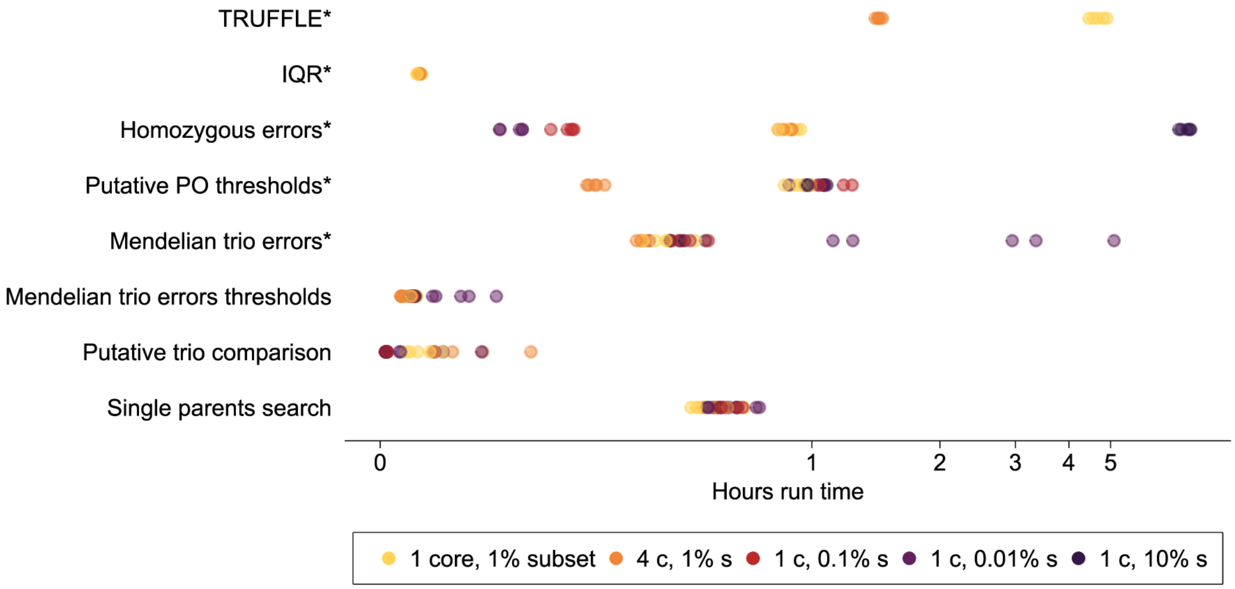

Figure S2: Runtimes in the simulated data sets for each step of *SPORE*. Colors indicate differently sized random subsets of the input variants to calculate homozygous mismatches and number of cores used (purple). Steps marked with an asterisk can optionally be skipped by providing/keeping the output from a previous *SPORE* run, for example if one only wants to change the APO value.

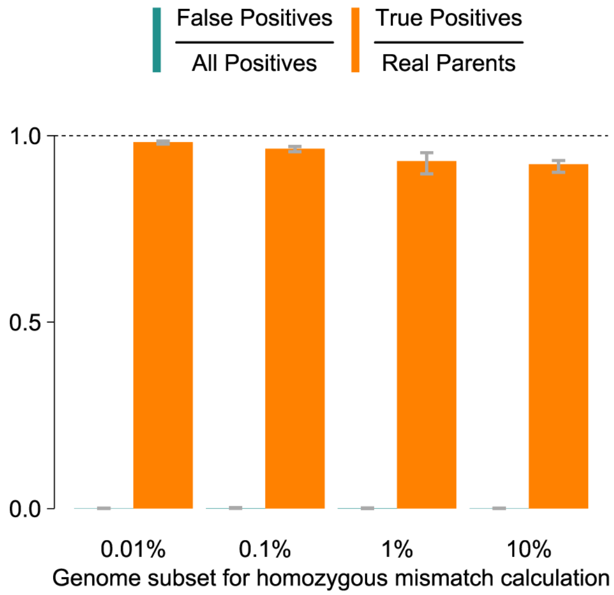

Figure S3: Varying genome subsets as a basis for calculation of homozygous mismatches in simulated pedigrees. Bars indicate the mean of five simulated pedigrees and the error bars show minimum and maximum values. Smaller values appear to improve the results, likely due to more individuals being included in putative trio comparisons. This is helpful when datasets are complete and the correct parents can be found.

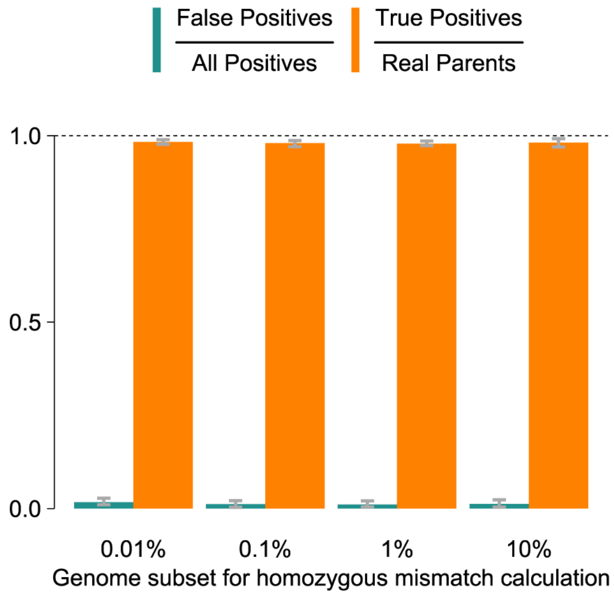

Figure S4: Varying genome subsets as a basis for calculation of homozygous mismatches in simulated pedigrees, randomly subset to 75% of individuals. Bars indicate the mean of five simulated pedigrees and the error bars show minimum and maximum values. In contrast to **Figure S3**, smaller values no longer improve results, because suboptimal pre-selection of putative PO will now lead to the inclusion of more wrong relationships, which impacts the quality of the chosen trios.

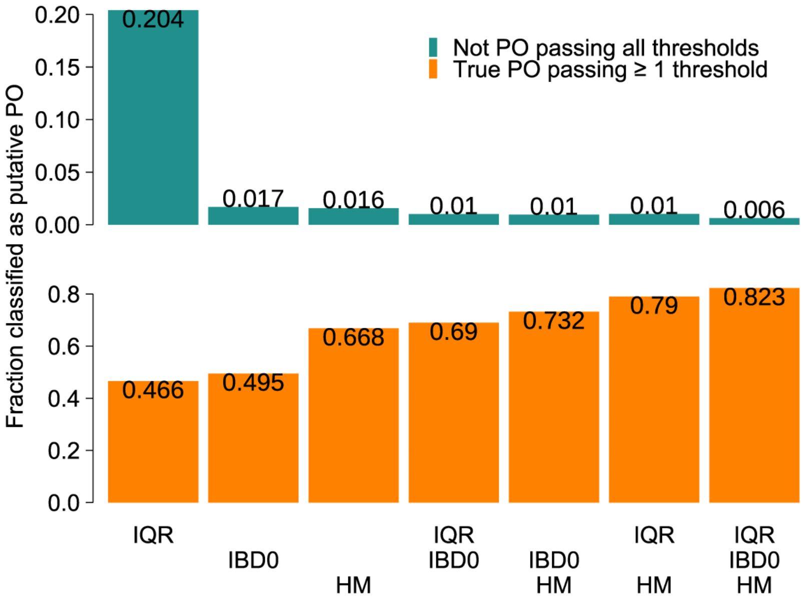

Figure S5: Fraction of relationships classified as putative parent-offspring (PO) in relation to which thresholds need to be passed. Top (green): fraction of relationships that are not true PO and pass all of the thresholds underneath each bar. This represents the false positives that would result from misclassification as PO in the third phase of *SPORE*. Bottom (orange): fraction of true PO relationships that pass at least one of the thresholds under each bar. This represents how well *SPORE* finds true PO in the first phase of the method. The figure is based on the simulated genotypes and APO=6.

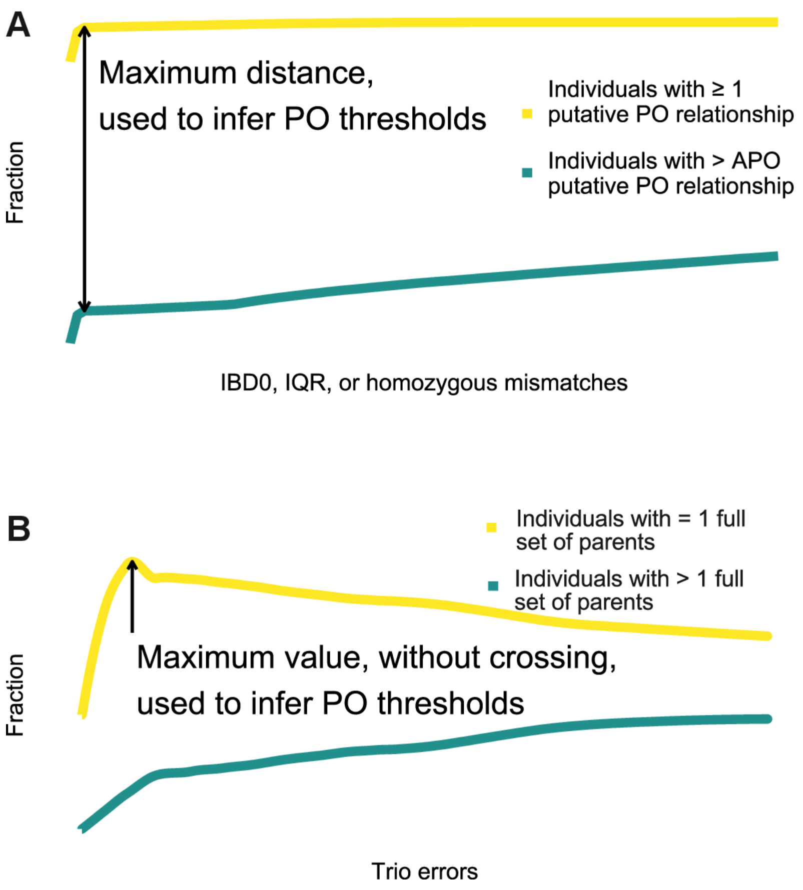

Figure S6: Example plots showing how automatic threshold calculation in *SPORE* works. The illustrations are based on averaged simulation data. **A)** Automatic thresholding for putative PO. In yellow, the fraction of individuals with ≥ 1 putative PO relationship if the x axis value was the threshold below which relationships are classified as PO. In green, the fraction of individuals with more PO relationships than the "assumed average PO relationships per individual," APO. A distance-drawing line highlights what *SPORE* selects as the threshold, the maximum distance between the two fractions. **B)** Automatic thresholding for trios. In yellow, the fraction of individuals that has exactly one full trio at the x-axis threshold. In green, the fraction of individuals that has more than one full trio. The threshold is then set to be the highest point on yellow as long as green has not crossed yellow.

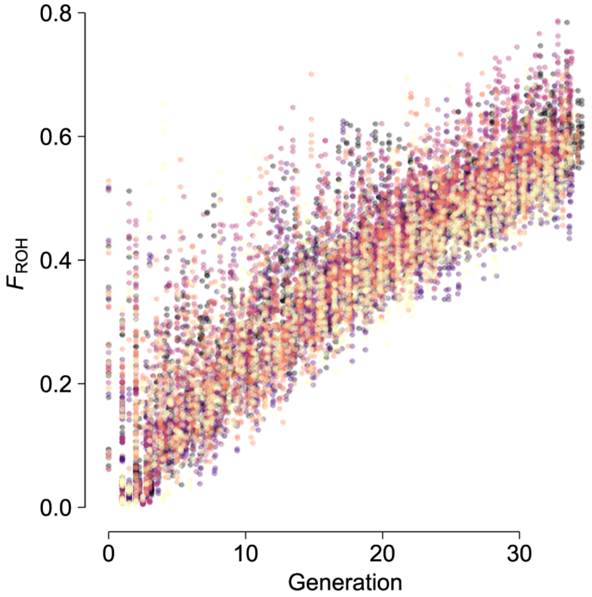

Figure S7: Individual inbreeding $F_{\mathbf{ROH}}$ values for all simulated individuals. The x axis represents the generation based on the true pedigree of the simulations. Colors denote the five unique simulated pedigrees.

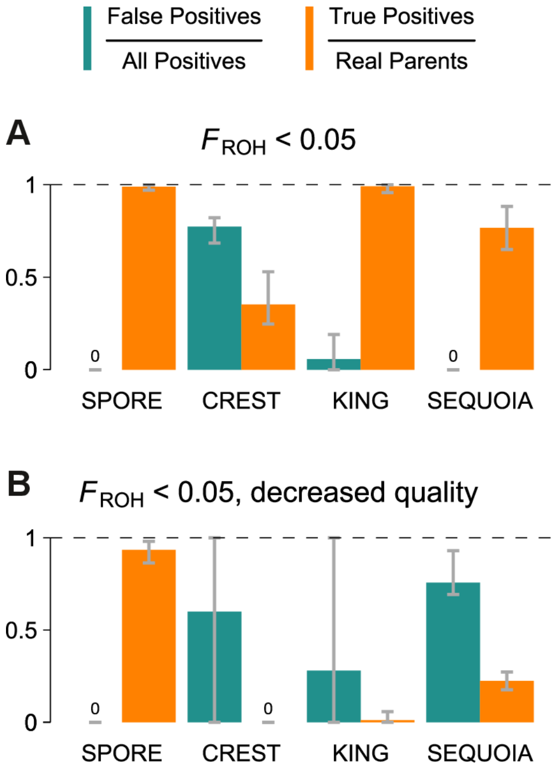

Figure S8: Parent-offspring inference performance with minimal inbreeding. Bars indicate the mean of five simulated pedigrees and the error bars show minimum and maximum values. These plots only include calls on individuals with $F_{\mathrm{ROH}} \leq 0.05$, which is only the case for 51 ± 25 parent-offspring relationships. **A)** Simulations with standard amount of genotyping errors. **B)** Simulations with increased genotyping errors. *SPORE*, *CREST*, and *SEQUOIA* calls are only evaluated as true if the inferred direction of the call (who is parent, who is offspring) is correct. *SEQUOIA* false positive calls only decrease by 3.4% (**B**) when direction is ignored.

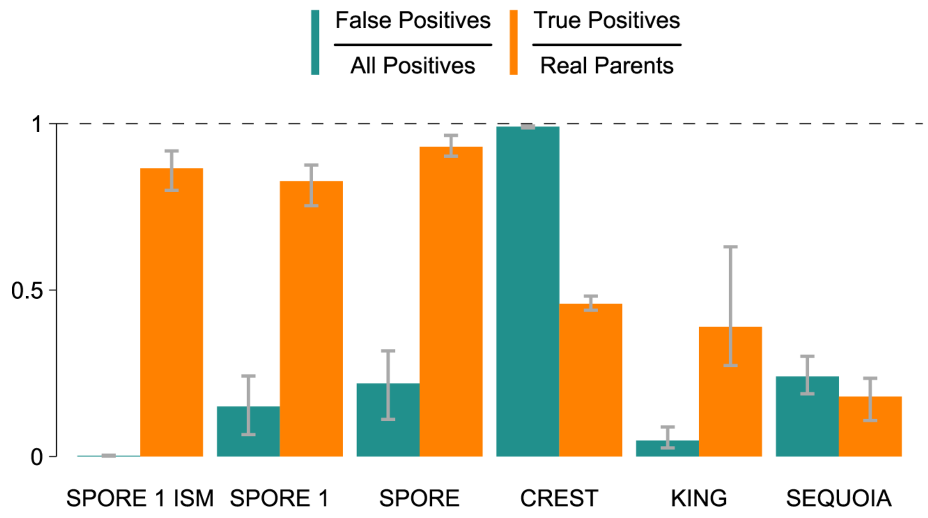

Figure S9: Parent-offspring inference performance in pedigrees where only 50% of the population was sampled (at random). Bars indicate the mean of five simulated pedigrees and the error bars show minimum and maximum values. "*SPORE* 1 ISM" shows results based on APO=1 with intermediate sampling mode; "*SPORE 1*" indicates results based on APO=1 and extensive sampling mode; "*SPORE*" uses APO=6 and extensive sampling mode as in all other plots. *SPORE*, *CREST*, and *SEQUOIA* calls are only evaluated as true if the inferred direction of the call (who is parent, who is offspring) is correct.

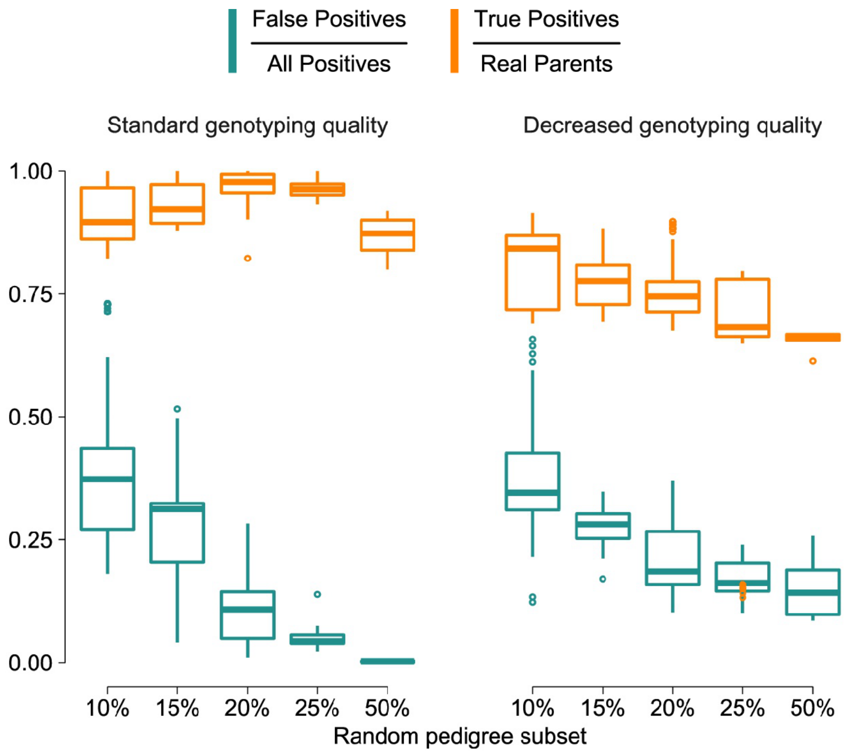

Figure S10: *SPORE* Performance in incomplete pedigrees. Results of intermediate sampling mode at APO=1 for different genotyping qualities (columns), with few errors in standard genotyping quality, based on five pedigrees sampled once each for the 50% condition and 11 times for all others.

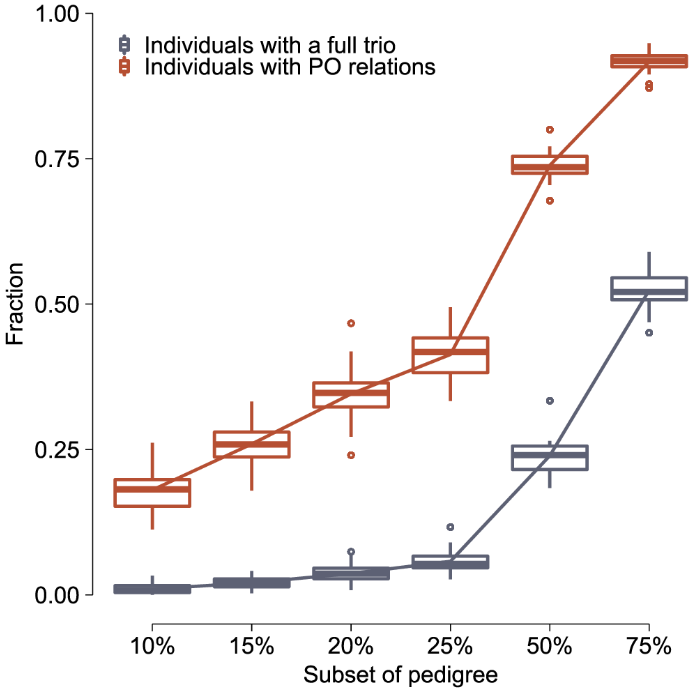

Figure S11: Fractions of individuals with at least one PO relationship (red) and a full trio (grey) in the analyzed random population subsets. Data based on five pedigrees sampled once each for the 50% condition and 11 times for all others. This figure is intended to help with the interpretation of other figures showing inference success for sub-sampled populations. Population subsets are created by randomly sampling a percentage of the population's individuals, irrespective of any traits or positions in the pedigree, intended to be comparable to sampling continuously from a population over time.

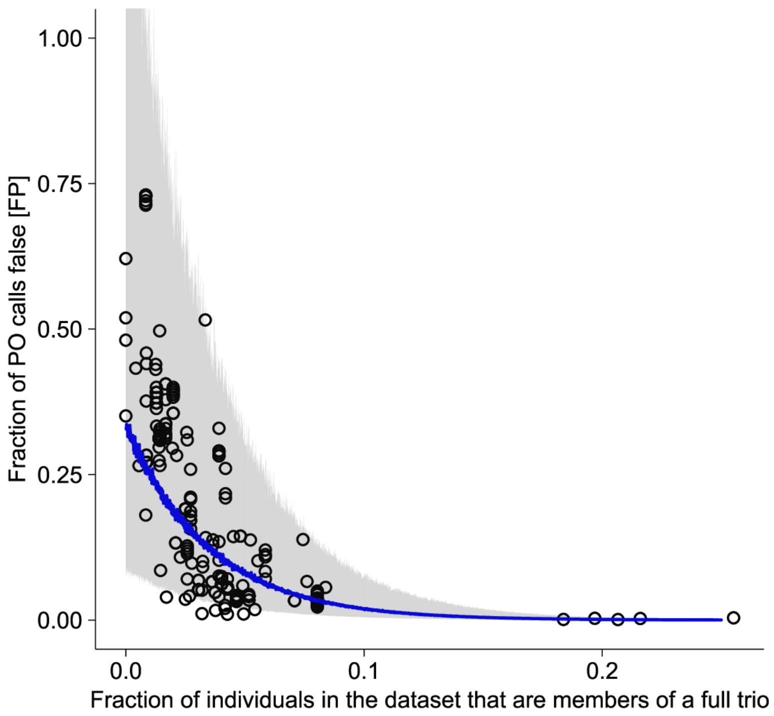

Figure S12: *SPORE*'s false positive rate under increasing presence of full trios in sampled datasets. The blue line indicates the median prediction of a linear mixed effect model with the random effect of simulation ID. The 95% confidence interval is presented in the shaded area. The data is based on random subsets of the simulated pedigrees with standard genotyping quality.

References

Abe, K. (2016). *Naturalsort: Natural ordering.* https://CRAN.R-project.org/package=naturalsort

Broad Institute. (2019). *Picard toolkit.* Broad Institute. http://broadinstitute.github.io/picard

Corbett-Detig, R., & Nielsen, R. (2017). A hidden Markov model approach for simultaneously estimating local ancestry and admixture time using next generation sequence data in samples of arbitrary ploidy. *PLOS Genet.*, *13*(1), 1–40. https://doi.org/10.1371/journal.pgen.1006529

Danecek, P., Auton, A., Abecasis, G., Albers, C. A., Banks, E., DePristo, M. A., Handsaker, R. E., Lunter, G., Marth, G. T., Sherry, S. T., McVean, G., & Durbin, R. (2011). The variant call format and VCFtools. *Bioinformatics*, *27*(15), 2156–2158. https://doi.org/10.1093/bioinformatics/btr330

Dimitromanolakis, A., Paterson, A. D., & Sun, L. (2019). Fast and accurate shared segment detection and relatedness estimation in un-phased genetic data via TRUFFLE. *Am. J. Hum. Genet.*, *105*(1), 78–88. https://doi.org/10.1016/j.ajhg.2019.05.007

Dowle, M., & Srinivasan, A. (2019). *Data.table: Extension of 'data.frame'.* https://cran.r-project.org/package=data.table

Druet, T., Alemu, S. W., Kadri, N. K., Faux, P., Harland, C., Charlier, C., & Caballero, A. (2020). An evaluation of inbreeding measures using a whole genome sequenced cattle pedigree. In *Dryad.* https://doi.org/10.5061/dryad.vx0k6djq8

Dumont, B. L., & Payseur, B. A. (2008). Evolution of the genomic rate of recombination in mammals. *Evolution*, *62*(2), 276–294. https://doi.org/10.1111/j.1558-5646.2007.00278.x

Ferrari, M., Lindholm, A. K., & König, B. (2019). Fitness consequences of female alternative reproductive tactics in house mice (*Mus musculus domesticus*). *Am. Nat.*, *193*(1), 106–124. https://doi.org/10.1086/700567

Hanghøj, K., Moltke, I., Andersen, P. A., Manica, A., & Korneliussen, T. S. (2019). Fast and accurate relatedness estimation from high-throughput sequencing data in the presence of inbreeding. *GigaScience*, *8*(5), giz034. https://doi.org/10.1093/gigascience/giz034

Jewett, E. M., McManus, K. F., Freyman, W. A., & Auton, A. (2021). Bonsai: An efficient method for inferring large human pedigrees from genotype data. *Am. J. Hum. Genet.*, *108*(11), 2052–2070. https://doi.org/10.1016/j.ajhg.2021.09.013

Johnson, E. C., Evans, L. M., & Keller, M. C. (2018). Relationships between estimated autozygosity and complex traits in the UK Biobank. *PLOS Genet.*, *14*(7), e1007556. https://doi.org/10.1371/journal.pgen.1007556

Kardos, M., Åkesson, M., Fountain, T., Flagstad, Ø., Liberg, O., Olason, P., Sand, H., Wabakken, P., Wikenros, C., & Ellegren, H. (2018). Genomic consequences of intensive inbreeding in an isolated wolf population. *Nat. Ecol. Evol.*, *2*(1), 124–131. https://doi.org/10.1038/s41559-017-0375-4

Kim, S., Scheffler, K., Halpern, A. L., Bekritsky, M. A., Noh, E., Källberg, M., Chen, X., Kim, Y., Beyter, D., Krusche, P., & Saunders, C. T. (2018). Strelka2: Fast and accurate calling of germline and somatic variants. *Nat. Methods*, *15*(8), 591–594. https://doi.org/10.1038/s41592-018-0051-x

Knowles, J. E., & Frederick, C. (2020). *merTools: Tools for analyzing mixed effect regression models*. https://cran.r-project.org/package=merTools

Ko, A., & Nielsen, R. (2017). Composite likelihood method for inferring local pedigrees. *PLOS Genet.*, *13*(8), e1006963. https://doi.org/10.1371/journal.pgen.1006963

König, B., & Lindholm, A. K. (2012). The complex social environment of female house mice (*Mus domesticus*). In M. Macholán, S. J. E. Baird, P. Munclinger, & J. Piálek (Eds.), *Evolution of the House Mouse* (pp. 114–134). Cambridge University Press. https://doi.org/10.1017/CBO9781139044547.007

Lemes, R. B., Nunes, K., Carnavalli, J. E. P., Kimura, L., Mingroni-Netto, R. C., Meyer, D., & Otto, P. A. (2018). Inbreeding estimates in human populations: Applying new approaches to an admixed Brazilian isolate. *PLOS ONE*, *13*(4), e0196360. https://doi.org/10.1371/journal.pone.0196360

Li, H. (2013). Aligning sequence reads, clone sequences and assembly contigs with BWA-MEM. *arXiv*, 1303.3997. http://arxiv.org/abs/1303.3997

Li, H. (2011). A statistical framework for SNP calling, mutation discovery, association mapping and population genetical parameter estimation from sequencing data. *Bioinformatics*, *27*(21), 2987–2993. https://doi.org/10.1093/bioinformatics/btr509

Li, H., Handsaker, B., Wysoker, A., Fennell, T., Ruan, J., Homer, N., Marth, G., Abecasis, G., & Durbin, R. (2009). The sequence alignment/map format and SAMtools. *Bioinformatics*, *25*(16), 2078–2079. https://doi.org/10.1093/bioinformatics/btp352

Li, J. H., Mazur, C. A., Berisa, T., & Pickrell, J. K. (2021). Low-pass sequencing increases the power of GWAS and decreases measurement error of polygenic risk scores compared to genotyping arrays. *Genome Res.*, *31*, 529–537. https://doi.org/10.1101/gr.266486.120

Liu, E. Y., Morgan, A. P., Chesler, E. J., Wang, W., Churchill, G. A., & Pardo-Manuel de Villena, F. (2014). High-resolution sex-specific linkage maps of the mouse reveal polarized distribution of crossovers in male germline. *Genetics*, *197*(1), 91–106. https://doi.org/10.1534/genetics.114.161653

Ma, L., O'Connell, J. R., VanRaden, P. M., Shen, B., Padhi, A., Sun, C., Bickhart, D. M., Cole, J. B., Null, D. J., Liu, G. E., Da, Y., & Wiggans, G. R. (2015). Cattle sex-specific

recombination and genetic control from a large pedigree analysis. *PLOS Genet.*, *11*(11), e1005387. https://doi.org/10.1371/journal.pgen.1005387

Martin, M., Patterson, M., Garg, S., O Fischer, S., Pisanti, N., Klau, G. W., Schöenhuth, A., & Marschall, T. (2016). WhatsHap: Fast and accurate read-based phasing. *bioRxiv*, 85050. https://doi.org/10.1101/085050

Narasimhan, V., Danecek, P., Scally, A., Xue, Y., Tyler-Smith, C., & Durbin, R. (2016). BCFtools/RoH: A hidden Markov model approach for detecting autozygosity from next-generation sequencing data. *Bioinformatics*, *32*(11), 1749–1751. https://doi.org/10.1093/bioinformatics/btw044

Ooms, J., & Csárdi, G. (2020). *Sys: Powerful and reliable tools for running system commands in R.* https://CRAN.R-project.org/package=sys

Peng, B., & Kimmel, M. (2005). simuPOP: A forward-time population genetics simulation environment. *Bioinformatics*, *21*(18), 3686–3687. https://doi.org/10.1093/bioinformatics/bti584

Qiao, Y., Sannerud, J. G., Basu-Roy, S., Hayward, C., & Williams, A. L. (2021). Distinguishing pedigree relationships via multi-way identity by descent sharing and sex-specific genetic maps. *Am. J. Hum. Genet.*, *108*(1), 68–83. https://doi.org/10.1016/j.ajhg.2020.12.004

R Core Team. (2018). *R: A language and environment for statistical computing.* R Foundation for Statistical Computing. https://www.r-project.org/

Rohatgi, A. (2021). *WebPlotDigitizer.* https://github.com/ankitrohatgi/WebPlotDigitizer

Seidman, D. N., Shenoy, S. A., Kim, M., Babu, R., Woods, I. G., Dyer, T. D., Lehman, D. M., Curran, J. E., Duggirala, R., Blangero, J., & Williams, A. L. (2020). Rapid, phase-free detection of long identity-by-descent segments enables effective relationship classification. *Am. J. Hum. Genet.*, *106*(4), 453–466. https://doi.org/10.1016/j.ajhg.2020.02.012

Staples, J., Qiao, D., Cho, M. H., Silverman, E. K., Nickerson, D. A., & Below, J. E. (2014). PRIMUS: Rapid reconstruction of pedigrees from genome-wide estimates of identity by descent. *Am. J. Hum. Genet.*, *95*(5), 553–564. https://doi.org/10.1016/j.ajhg.2014.10.005

Szpiech, Z. A., Xu, J., Pemberton, T. J., Peng, W., Zöllner, S., Rosenberg, N. A., & Li, J. Z. (2013). Long runs of homozygosity are enriched for deleterious variation. *Am. J. Hum. Genet.*, *93*(1), 90–102. https://doi.org/10.1016/j.ajhg.2013.05.003

Talebi, R., Szmatoła, T., Mészáros, G., & Qanbari, S. (2020). Runs of homozygosity in modern chicken revealed by sequence data. *G3-Genes Genom Genet*, *10*(12), 4615–4623. https://doi.org/10.1534/g3.120.401860

Tian, D., Turner, B. J., & Martin, C. H. (2021). Severe inbreeding and gene loss in the historical and extant population of the critically endangered Devils Hole pupfish. *bioRxiv*, 2021.08.13.456274. https://doi.org/10.1101/2021.08.13.456274

Venables, W. N., & Ripley, B. D. (2002). *Modern Applied Statistics with S* (Fourth). Springer. https://www.stats.ox.ac.uk/pub/MASS4/

Waples, R. K., Albrechtsen, A., & Moltke, I. (2019). Allele frequency-free inference of close familial relationships from genotypes or low-depth sequencing data. *Mol. Ecol.*, *28*(1), 35–48. https://doi.org/10.1111/mec.14954

Wellmann, R. (2021). *optiSel: Optimum contribution selection and population genetics.* https://CRAN.R-project.org/package=optiSel

Whalen, A., Gorjanc, G., & Hickey, J. M. (2019). Parentage assignment with genotyping-by-sequencing data. *J. Anim. Breed. Genet.*, *136*(2), 102–112. https://doi.org/10.1111/jbg.12370

Wickham, H. (2019). *Stringr: Simple, consistent wrappers for common string operations.* https://cran.r-project.org/package=stringr

Wickham, H. (2016). *ggplot2: Elegant Graphics for Data Analysis.* Springer-Verlag. https://doi.org/10.1007/978-3-319-24277-4

Wickham, H., François, R., Henry, L., & Müller, K. (2019). *Dplyr: A grammar of data manipulation.* https://cran.r-project.org/package=dplyr

Wickham, H., Hester, J., & François, R. (2018). *Readr: Read rectangular text data.* https://cran.r-project.org/package=readr

Wickham, H., & RStudio. (2021). *Tidyr: Tidy messy data.* https://CRAN.R-project.org/package=tidyr
